# Stabilization of Transcription Factor, HIF-1α by Prolylhydroxylase 1 Knockout Reduces Cardiac Injury After Myocardial Infarction in Mice

**DOI:** 10.3390/cells14060423

**Published:** 2025-03-13

**Authors:** Mahesh Thirunavukkarasu, Seetur R. Pradeep, Babatunde Oriowo, Sue Ting Lim, Monica Maloney, Shayan Ahmed, Nicole Taylor, David M. Russell, Pavayee Socrates, Ethan Batko, Matan Berkovsky, John Alexander Palesty, Nilanjana Maulik

**Affiliations:** 1 Molecular Cardiology and Angiogenesis Laboratory, Department of Surgery, University of Connecticut School of Medicine, UConn Health, Farmington, CT 06030, USA; mthirunavukkarasu@uchc.edu (M.T.); srpradeep2018@gmail.com (S.R.P.); baoriowo@gmail.com (B.O.); limsueting@gmail.com (S.T.L.); momaloney@uchc.edu (M.M.); ahmedshayan117@gmail.com (S.A.); nictaylor@uchc.edu (N.T.); russel@uchc.edu (D.M.R.); socrates@uchc.edu (P.S.); ethanbatko@gmail.com (E.B.); matan.berkovsky@einsteinmed.edu (M.B.); 2 Stanley J. Dudrick, Department of Surgery, Trinity Health of New England—Saint Mary’s Hospital, Waterbury, CT 06706, USA; alexander.palesty@trinityhealthofne.org

**Keywords:** prolyl hydroxylase-1, myocardial infarction, HIF-1α, cardiac functions, pathway analysis

## Abstract

Inhibition of HIF-prolyl hydroxylases (PHD1, PHD2, and PHD3) causes the stabilization of hypoxia-inducible factor-1α and -2α (HIF-1α and HIF-2α) to regulate various cell signaling pathways. Hypoxia-inducible factor (HIF) is crucial in regulating signal responses mediated by hypoxia. HIF regulates the transcription of many genes involved in the response to hypoxia and ischemic insult. Our current work investigates the protective effects of PHD1 knockout in mice against myocardial infarction. **Study Design:** Myocardial infarction (MI) was induced by left anterior descending coronary artery (LAD) ligation (8–12-week-old mice) in both wild-type (WT) and PHD1 knockout (PHD1^−/−^) mice. WT sham (S) and PHD1^−/−^S group mice underwent surgery without LAD ligation. Thirty days post-surgery, cardiac functions were measured by echocardiogram. Mice in all the groups were euthanized at various time points for tissue collection post-MI 8 h (gel shift and microarray analysis), 4 days (Western blot analysis), 7 days (blood vessel density), or 30 days (histological analysis). For microarray analysis, WTMI and PHD1^−/−^MI group mices’ heart tissue was used for RNA isolation, then hybridization to a GeneChip™ Mouse Gene 1.0 ST Array as per the manufacturer’s instructions. Bioinformatic analysis was performed using the transcriptome analysis console (TAC) to generate a list of differentially regulated genes, followed by ingenuity pathway analysis. **Results:** The study findings revealed a significant increase in vessel density (capillary and arteriolar density) in the PHD1^−/−^MI mice compared to those with WTMI. The echocardiographic examination demonstrated that the PHD1^−/−^MI mice group had an increased ejection fraction and fractional shortening than the WT mice 30 days post-MI. HIF-1α DNA binding activity was higher in PHD1^−/−^MI mice than in WTMI. The Western blot analysis showed a significant increase in the expression of HSPA12B in the PHD1^−/−^MI compared to WTMI mice. Bioinformatic analysis using TAC software, Version 4.0.2.15 (1.5 fold, *p* < 0.05) showed 174 differentially regulated genes. **Conclusions:** In conclusion, our study showed PHD1 knockout activates several important molecules and signaling pathways, resulting in increased angiogenesis and cardioprotection against myocardial infarction.

## 1. Introduction

Myocardial infarction (MI) caused by coronary artery disease is associated with significant mortality [1]. Clinical trials for the treatment of MI have provided irregular results so far; therefore, the induction of clinically useful therapeutic angiogenesis remain challenging [2,3,4]. Studies are necessary to understand the molecular mechanism of angiogenesis to develop therapeutics. There is a discrepancy related to the molecular signaling that increases angiogenesis and reduces ventricular remodeling in MI animals. MI-mediated downregulation of growth factors and increased oxidative stress led to impaired angiogenesis [5]. This creates a redox imbalance, which triggers stress-related complications in the system. The PHDs (prolyl hydroxylases) PHD1, PHD2 and PHD3 target HIF-1α for degradation. Hypoxia or ischemia inactivates PHDs and results in HIF-1α accumulation in the system [6,7,8].

Our previous findings [9] reported that deleting PHD1 and PHD-3 increased angiogenesis and blood perfusion in the murine hind-limb ischemia model by upregulating proangiogenic factor VEGF and HIF-1α. In addition, there was increased vessel density and increased protein expression, of BCl-2, in the PHD1 and PHD-3 ischemic limbs. In another study, PHD1 global knockout mice subjected to the ex vivo ischemia–reperfusion model (I/R model) also demonstrated preserved cardiac function in PHD1 mice compared to the corresponding wild-type controls [10]. In addition, our study with cardiac-specific PHD-2 knockout mice subjected to MI also demonstrated increased angiogenesis and heart function when compared to the wild-type controls [11]. When there is reduced oxygen (hypoxic or ischemic situation) in the system, both the subunits (HIF-1α and HIF-2α) move from the cytosol to the nucleus, where they dimerize with their respective subunits (HIF-1α and HIF-2β) to form active transcription factor. This transcription factor’s active form controls the regulation of hundreds of cellular genes, resulting in ischemic tissue regeneration, cell survival, and repair [12]. The HIF-1α subunit controls HIF activity, while HIF-hydroxylases regulate the oxygen-dependent hydroxylation of the HIF-1α subunit to limit its half-life. HIF-1α is hydroxylated under normoxic conditions by PHDs. Hydroxylation by PHDs enables HIF-1α proteasomal degradation via ubiquitination [13,14]. Nearly, all PHDs have almost similar functions; however, they appear to have dissimilar specificities for various hydroxylation sites [15,16]. HIF hydroxylases are the primary oxygen sensors in our system [15,16,17].

According to reports, a PHD inhibitor named GSK360A taken orally can protect the ischemic heart following myocardial infarction and increase HIF-1α signaling [18]. Additionally, PHD1 knockdown decreased mouse liver ischemia–reperfusion injury [19]. Also, knockout of PHD1 stimulates skeletal muscle by altering base-level metabolism, thereby increasing hypoxic tolerance [20]. Our previous study demonstrated that PHD1 knockout (PHD1^−/−^) improved myocardial function after ischemia–reperfusion injury. The mechanism was likely due to HIF-1α stabilization followed by angiogenic protein expression [21]. We also explored increased VEGF expression, angiogenic response, and vascularization in the murine hind-limb ischemia (HLI) model with PHD1^−/−^ and PHD-3^−/−^ mice compared to its wild-type counterpart [9]. However, further research or investigation is warranted using in vivo models of mouse MI and identifying differential gene expression to develop and understand the molecular mechanisms that underlie the knockout of PHD1.

Our present approach in regulating HIF-1α by inhibiting PHD1 may have been always associated with significant mortality strategic therapeutic potential for treating ischemic disorders, particularly in the heart. We will approach gene deletion (loss of function) technology to identify the molecular signaling pathway and identify the target molecules that participate in angiogenesis, cell survival, and cell differentiation. This will be performed via the GeneChip™ Mouse Gene 1.0 ST Array (Thermo Fisher Scientific, Agawam, MA, USA) by exposing both wild-type controls and PHD1 global knockout mice to MI.

## 2. Materials and Methods

### 2.1. Animal Studies

This study was performed per the principles of laboratory animal care formulated by the National Society for Medical Research and described in the *Guide for the Care and Use of Laboratory Animals* prepared by the National Academy of Sciences and published by the National Institutes of Health (Publication No. 85-23, revised 1985). The animal care committee of the University of Connecticut Health (Farmington, CT, USA) examined and approved the animal protocol. Eight-to-twelve-week-old male and female HIF-prolyl hydroxylase-1 knockout (PHD1^−/−^) mice and respective wild-type (WT) mice were used in this study. They were generated as described in a previous study [22] and were a kind gift from Dr. Fong GH, Vascular Biology, University of Connecticut Health Center (Farmington, CT, USA). PHD1^−/−^ mice genotyping was confirmed using polymerase chain reaction (PCR) on pure DNA from tail samples [21].

### 2.2. Experimental Design

Eight-to-twelve-week-old male and female PHD1^−/−^ mice and WT mice were exposed to either sham surgery or experimental MI surgery. The mice were split into four total experimental groups: (1) wild-type sham (WTS), (2) HIF-prolyl hydroxylase-1 knockout sham (PHD1^−/−^S), (3) wild-type MI (WTMI), (4) HIF-prolyl hydroxylase-1 knockout MI (PHD1^−/−^MI).

**Figure 1:**
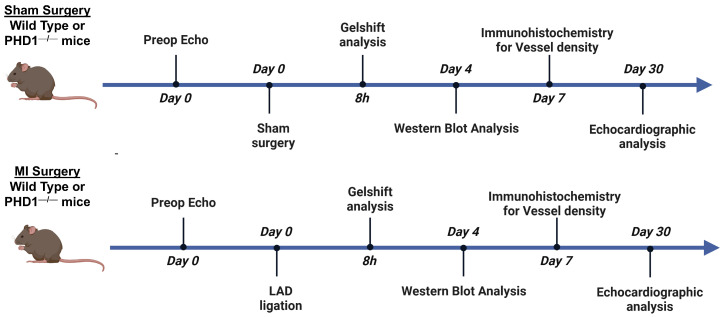
Graphical representation of the timeline in each group of mice. On day 0, all groups underwent preoperative echocardiography. Wild-type mice and PHD1^−/−^ mice underwent sham surgery or LAD ligation. Samples are collected in all groups at 8 h for gel shift analysis, day 4 for Western blot analysis, day 7 for immunohistochemistry, and day 30 for echocardiographic analysis. Created with BioRender (https://BioRender.com).

MI was induced by permanent left anterior descending (LAD) coronary artery ligation. Sham groups underwent a thoracotomy without permanent ligation of the LAD. Myocardial infarction groups underwent a thoracotomy with permanent ligation of the LAD. Left ventricular tissue was collected for RNA isolation and microarray analysis eight hours post-MI. Four days post-MI, tissue samples were collected for Western blot analysis. Vessel density analysis was performed seven days after surgery. Echocardiography was performed, followed by immunohistochemical analysis 30 days after surgery. Experimental protocol is shown in Figure 1.

### 2.3. Surgical Procedure

Ketamine (100 mg/kg, ip) and xylazine (10 mg/kg, ip) were used as anesthesia to sedate the mice. A 22-G IV catheter was used for oral intubation, and a rodent respirator (Harvard Apparatus, Holliston, MA, USA) was used for ventilation. Mice were allowed to reach full sedation. The mice were positioned supine with slight right lateral decubitus angulation and secured with tape to a firm board. The chest was then prepared for surgery. Chest hair was removed with Nair and a cotton swab. The exposed skin was cleaned with sterile betadine and sterile alcohol swabs. A left lateral thoracotomy was performed. Permanent LAD ligation to create myocardial infarction (MI) was carried out with 8–0 polypropylene suture. The lungs were inflated by positive end-expiratory pressure, and the chest was closed with a running 6-0 polypropylene suture. The skin was closed with a running 5-0 vicryl suture. After surgery, the analgesic buprenorphine (0.1 mg/kg, sc) was given. The animals were weaned from the respirator and placed on a heating pad for recovery [11].

### 2.4. RNA Isolation

After completion of the treatment protocols, left ventricular tissue was quickly frozen in liquid nitrogen and stored at −80 °C. Frozen left ventricles (n = 4/group for microarray analysis) were homogenized, and DNA-free total RNA was extracted using the TRIzol kit (Catalog # 15596026, Invitrogen, Agawam, MA, USA) and further purified using RNeasy RNA isolation kit (Qiagen, Valencia, CA, USA), according to the manufacturer’s instruction. RNA purity and integrity were evaluated by denaturing gel electrophoresis on Agilent 2100 Bioanalyzer chips (Agilent Technologies, Palo Alto, CA, USA) [11].

### 2.5. Microarray Analysis

For microarray analysis, mouse heart tissues were collected from WTMI and PHD1^−/−^MI groups. The tissues were used for RNA isolation, then hybridization to the GeneChip™ Mouse Gene 1.0 ST Array as per the manufacturer’s instructions. Bioinformatic analysis was performed using the transcriptome analysis console (TAC) to generate the list of differentially regulated genes, followed by ingenuity pathway analysis. The genes that were found were mapped to genetic networks that were accessible via the Ingenuity database. Subsequently, these genes were graded based on their respective scores. A *p*-value of 0.05 was established as the significance level. The PANTHER classification system (https://pantherdb.org/, 27 February 2025) was used for focused pathway analysis by uploading both upregulated and downregulated list separately [23]. Also, gene set enrichment analysis was performed using online available software ShinyGO 0.77, version 0.77 (http://bioinformatics.sdstate.edu/go77/) [24], and the results are provided in Figure 7C.

### 2.6. Gel Shift Analysis

Electrophoretic mobility shift assay (EMSA)/gel shift was performed as described in our earlier publication [21].

### 2.7. Western Blot Analysis

Animals were anesthetized to collect left ventricular peri-infarct cardiac tissues 4 days after myocardial infarction (MI) and were used for gel electrophoresis and Western blot analysis as described previously [25,26]. SDS-PAGE electrophoresis was performed, and the separated proteins in the gel were transferred to a polyvinylidene difluoride (PVDF) membrane (Cat # IPVH00010, Millipore, Billerica, MA, USA). The membrane was then blocked using 5% milk solution for 1 h, followed by incubation with HSPA12B primary antibody (Cat # Ab4110/Gift from Dr. Zhihua Han (Produced in ProSci Inc., Poway, CA, USA) [27]. For IRS2 protein expression, we performed the immunoprecipitation immunoblotting (IP-IB) method. Immunoprecipitation was performed using IRS2 antibody (Cat #: Sc-493; Santa Cruz Biotechnology, Inc., Dallas, TX, USA) [27]. Protein expression was detected as described in our previous publications [11].

### 2.8. Immunohistochemical Analysis to Determine Capillary and Arteriolar Density in Murine Cardiac Tissue

As described in our previous publication, capillary and arteriolar density were performed in heart tissue 7- and 30-days post-MI, respectively. A goat polyclonal anti-CD31 antibody (Cat. # AF3628; R&D Systems, Wallingford, CT, USA) was used to stain capillaries for capillary density analysis. For arteriolar density staining, α-smooth muscle actin (1:100 in PBS; Cat. #: ab7817, Abcam Inc., Waltham, MA, USA) antibody was used to stain arterioles. Both capillary and arteriolar density were represented as counts per mm^2^ [11]. The sections were examined and photographs were captured using a laser scanning confocal microscope (LSM 510 meta) for arteriolar density and Olympus BH2 microscope for capillary density.

### 2.9. Echocardiographic Evaluation of Cardiac Morphology and Function

As described in previous studies, an echocardiogram was performed preoperatively and on postoperative day 30. Eight-to-twelve-week-old male and female PHD1^−/−^ mice and WT mice were sedated using 2% isoflurane and secured in the supine position on a heated, custom-designed pad associated with the echocardiographic ultrasound machine set up [21]. The chest was prepared for ultrasonography. The chest hair was removed with Nair and a cotton swab. Two-dimensional echocardiography was performed using Vevo 770, Visual-Sonics Inc. ultrasound machine, and heart images were captured and analyzed [11].

## 3. Statistics

An unpaired *t*-test was employed to assess the differences between the two groups, and for multiple-group comparison, a one-way ANOVA was followed by Tukey’s post hoc analysis. Values are mean ± standard error of the mean (SEM). Using GraphPad Prism software, version 9 (GraphPad Prism version 10.2.3), statistical analysis was carried out [11].

## 4. Results

### 4.1. Baseline Cardiac Function in PHD1^−/−^ Mice

Echocardiography was performed on the WT mice and PHD1^−/−^ mice at baseline, and various parameters were analyzed. When compared to the WT mice, the PHD1^−/−^ mice showed no significant difference in mean HR (446.8 ± 28.46 vs. 447 ± 22 beats per minute), ejection fraction (67.54 ± 3.36% vs. 69 ± 3.11%), and fractional shortening (36.99 ± 2.542% vs. 38.15 ± 2.595%). There was no significant difference in the left ventricular diastolic interventricular diameter (LVIDd) between the two groups at baseline (3.558 ± 0.1465 mm vs. 3.534 ± 0.007 mm) as well as left ventricle interventricular diameter at systole (LVID;s) (2.242 ± 0.135 mm vs. 2.182 ± 0.093 mm) (n = 9–12) [21].

### 4.2. PHD1^−/−^ Mice Show Increased Capillary Density and Arteriolar Density Compared to WT Mice After MI

**Figure 2:**
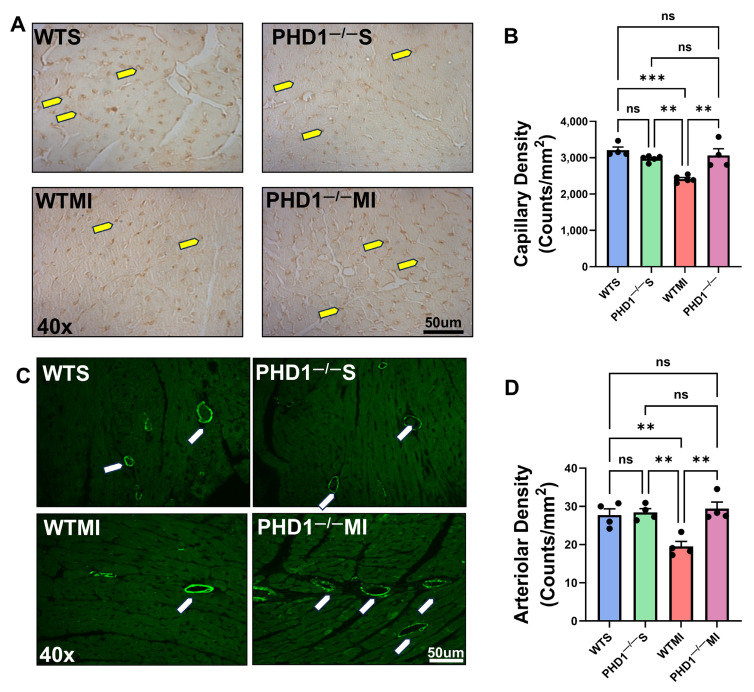
(**A**) Representative microphotographs of immunohistochemistry analysis demonstrating capillary density in cardiac tissue for each group of mice. There is a significant increase in capillary density in PHD1^−/−^ MI mice compared to WTMI mice. (**B**) The capillary density in each group of murine cardiac tissue is represented in bar graph form (n = 4–5). ns represents no difference in capillary density between the groups, while asterisks represent the degree of significance between the two groups. (**C**) Representative microphotographs of immunofluorescence study demonstrating capillary density in cardiac tissue for each group of mice. There is a significant increase in arteriolar density in PHD1^−/−^ MI mice compared to WTMI mice. (**D**) Arteriolar density in murine cardiac tissue of each group is represented in bar graph form (n = 4). Values are mean ± SEM. ns represents no difference in capillary density between the groups, while asterisks represent the degree of significance between the two groups (** *p* < 0.01; *** *p* < 0.001). Yellow arrow denotes capillaries and white arrow denotes arterioles.

Cardiac tissue samples were taken from the at-risk areas of each group of mice on postoperative day seven to determine capillary and arteriolar density via immunohistochemistry. No significant difference was found between the capillary density of WTS mice and PHD1^−/−^ S mice (3209 vs. 2975 counts/mm^2^, *p* = 0.4673). Our results demonstrate the preservation of capillary density in the cardiac tissue in PHD1^−/−^ MI mice compared to PHD1^−/−^ S mice (3066 ± 182 vs. 2975 ± 36.6, *p* = 0.9856). In contrast, there was a significant reduction in capillary density in the WTMI mice compared to the WTS mice (2415 ± 41.2 vs. 3209 ± 87.26, *p* = 0.0002). Notably, although there was no significant difference between the WT mice and PHD1^−/−^ mice after undergoing a sham procedure, there was a significant difference in capillary density following MI in the two groups, with the PHD1^−/−^ MI mice showing maintenance of the capillary density in the at-risk area. In contrast, the capillary density in the WTMI myocardium was significantly decreased (3066 ± 182 vs. 2415 ± 41.2, *p* = 0.0014) (n = 4–5) (Figure 2A,B).

Similarly, we analyzed the arteriolar density in the at-risk area of the myocardial tissues and found results correlating to capillary density. We found no significant difference between the arteriolar density of the WTS mice and PHD1^−/−^ S mice (27.75 ± 1.594 vs. 28.41 ± 1.008 counts/mm^2^, *p* = 0.9998). Our results again demonstrate the preservation of arteriolar density in the cardiac tissue in PHD1^−/−^ MI mice when compared to PHD1^−/−^ S mice (29.41 ± 1.726 vs. 28.41 ± 1.008, *p* = 0.9975). Conversely, there was a significant reduction in arteriolar density in the WTMI mice when compared to the WTS mice (19.51 ± 1.329 vs. 27.75 ± 1.594, *p* = 0.0097). After undergoing sham procedures, there was no significant difference in arteriolar density between the WT mice and PHD1^−/−^ mice, in contrast to the significant difference observed following MI in the two groups, with the PHD1^−/−^ MI mice showing maintenance of the arteriolar density in the at-risk area. However, the WTMI mice suffered a significant reduction in arteriolar density (29.41 ± 1.726 vs. 19.51 ± 1.329, *p* = 0023) (Figure 2C,D).

### 4.3. The Cardiac Function of PHD1^−/−^ Mice Outperforms the Cardiac Function of WT Mice at 30 d Following MI

**Figure 3:**
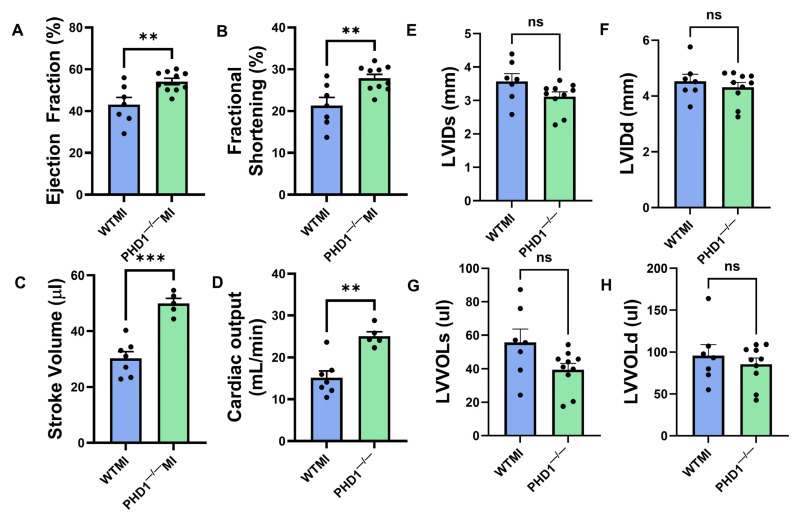
Graphical representation of various parameters in the echocardiographic analysis of the WTMI mice and PHD1^−/−^ mice, showing significant differences in (**A**) ejection fraction, (**B**) fractional. shortening, (**C**) stroke volume, (**D**) cardiac output, (**E**) left ventricular internal diameter systole, (**F**) left ventricular internal diameter diastole, (**G**) left ventricular volume systole, (**H**) left ventricular volume diastole (n = 5–7). Values are mean ± SEM (** *p* < 0.01; *** *p* < 0.001). Asterisks represent the degree of significance between the two groups (ns = not significant).

Our study shows a significantly preserved ejection fraction in PHD1^−/−^ mice when compared to WT mice (54.17 ± 1.516 vs. 43.11 ± 3.479%, *p* = 0.0054) at 30 days following MI, as well as a significantly preserved fractional shortening (27.85 ± 0.953 vs. 21.32 ± 1.957, *p* = 0.0049). The stroke volume of the PHD1^−/−^ mice was also superior to the WT mice following MI (49.9 ± 1.801 vs. 30.33 ± 2.371 µL, *p* = 0.0001) as well as cardiac output (25.04 ± 1.091 vs. 15.14 ± 1.64 mL/min, *p* = 0.0010; n = 5–7). These results demonstrate the role of PHD1 knockout in preserving cardiac function following MI (Figure 3A–H).

## 5. Microarray Results

**Figure 4:**
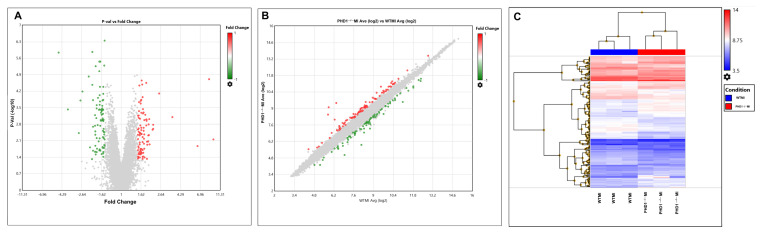
(**A**) Volcano plot and (**B**) scattered plot. Red dots represent positive fold change, while green dots represent negative fold change. (**C**) Heat map provides overall information of differentially regulated genes in a pictorial representation format of WTMI and PHD1^−/−^MI comparisons. Heat map showing differentially expressed genes in WT and PHD1^−/−^ MI groups. The blue boxes indicate downregulated expression of genes, whereas the red boxes indicate upregulated expression of genes. The intensity of the red shade represents the degree of upregulation, while the degree of downregulation is the intensity of the blue shade.

Microarray analysis was performed to determine differentially expressed genes in wild-type and PHD1^−/−^ knockout mice after MI. TAC software, Version 4.0.2.15 was used for bioinformatic analysis (1.5-fold, *p* < 0.05), which revealed 174 differentially regulated genes, of which 89 were upregulated (Table 1) and 85 were downregulated genes (Table 2). A volcano plot, scatter plot, and heat map analysis were generated, and the pictures are shown in Figure 4A, Figure 4B, and Figure 4C, respectively. Gene lists from the volcano plot analysis were further used for downstream pathway analysis, as described below.

**Figure 5:**
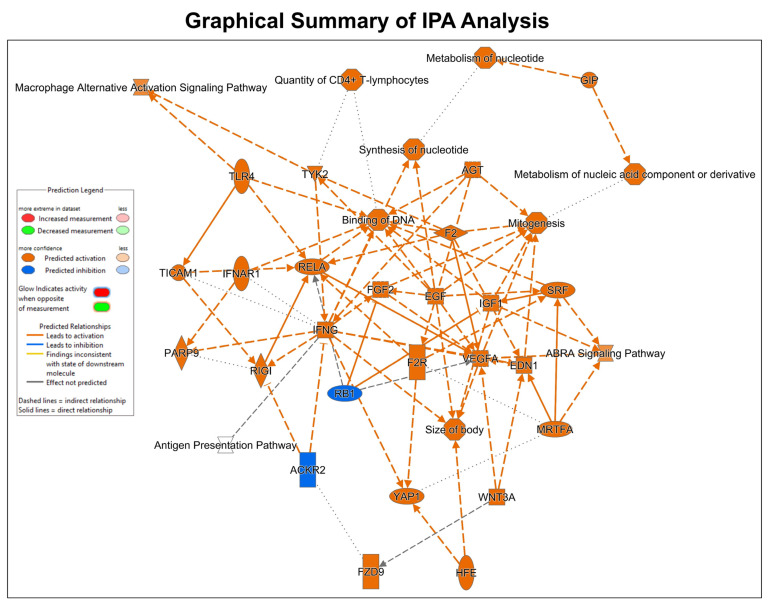
This picture represents the overall summary of the Ingenuity pathway analysis.

**Figure 6:**
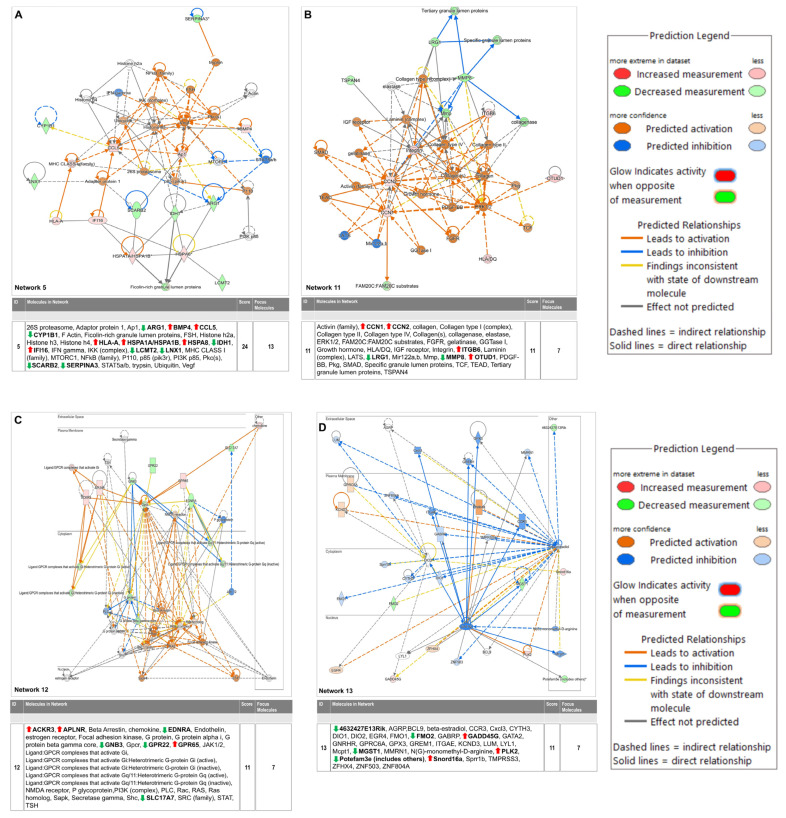
Selected critical networks in relation to (**A**) Cell Death and Survival, Cellular Compromise, Embryonic Development (Network 5), (**B**) Cellular Assembly and Organization, Cellular Movement, Hematological System Development and Function (Network 11), (**C**) Cardiovascular Disease, Congenital Heart Anomaly, Developmental Disorder (Network 12) and (**D**) Connective Tissue Development and Function, Embryonic Development, Organismal Development (Network 13). * Duplicates—Gene/Protein/Chemical identifiers marked with an asterisk indicate that multiple identifiers in the dataset file map to a single gene/chemical in the Global Molecular Network.

The differentially regulated 174 genes were subsequently imported into the Ingenuity pathway analysis software (version 24.0.2). The graphical summary IPA is given in Figure 5, which shows several key signaling pathways, out of which the key pathways related to myocardial angiogenesis are VEGF signaling, YAP1 signaling, WNT signaling, IGF signaling, FGF signaling, EGF signaling, and TLR4 signaling. Also, the in-depth analysis of the genes using the tool network analysis in IPA revealed 13 gene interaction networks. Among these networks, one of the most significant (network 11) is linked to cardiovascular disease. The network exhibited clustering with multiple vital genes, such as ACKR3, APLNR, Beta Arrestin, chemokine, EDNRA, Endothelin, estrogen receptor, Focal adhesion kinase, G protein, PI3K (complex), and others. Network 5 concerning cell death and survival included genes such as 26S proteasome, Adaptor protein 1, Ap1, ARG1, BMP4, CCL5, CYP1B1, F Actin, Ficolin-rich granule lumen proteins, FSH, Histone h2a, Histone h3, Histone h4, HLA-A, HSPA1A/HSPA1B, HSPA8, IDH1, IFI16, IFN gamma, IKK (complex), LCMT2, LNX1, MHC CLASS I (family), MTORC1, NFkB (family), P110, p85 (pik3r), PI3K p85, Pkc(s), SCARB2, SERPINA3, STAT5a/b, trypsin, Ubiquitin, and Vegf. The IPA pathway analysis revealed a significant association between cardiac fibrosis with a set of 10 molecules (Airn, ARG1, ATF3, CCN2, CYP1B1, EGR1, HAND2, LCN2, PFKFB1, SCN5A) in the PHD1^−/−^MI group (*p* = 0.0000382). The complete information of networks clustered by IPA is provided in Table 3, which contains 13 networks. IPA was used to construct a graphical network of important networks 5 (Figure 6A), 11 (Figure 6B), 12 (Figure 6C) and 13 (Figure 6D). Table 4 lists related cardiac fibrosis genes and their role in fibrosis. The list of (anticipated) activated or inhibited regulators that can raise, or lower functional molecules downstream is displayed in Table 5. Ingenuity pathway analysis identified several anti-inflammatory diseases and several anti-inflammatory molecules, as listed in Table 6. In addition, Table S1 provides additional information related to the canonical pathway. Several metabolic and cell signaling pathways are listed in this table. Table S1 shows both key biological and molecular roles of proteins and chemicals of interest. Table 7 shows the results of gene enrichment analysis of top 30 pathways identified.

### 5.1. Gene Ontology and Gene Set Enrichment Analysis

**Figure 7:**
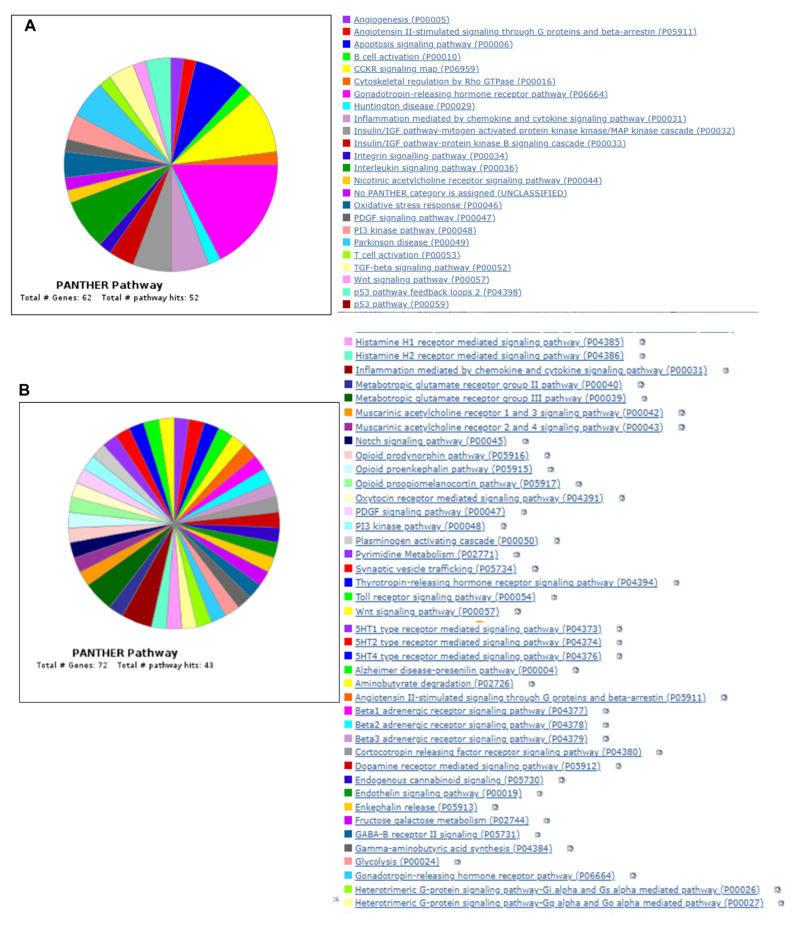

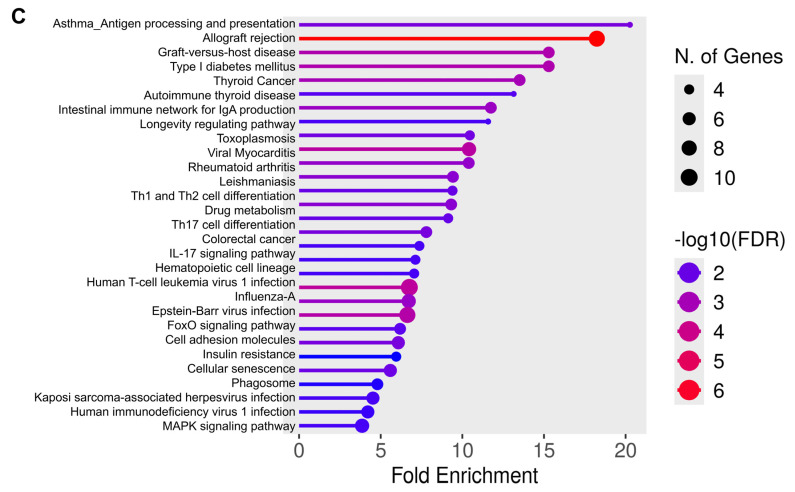
Venn diagram showing Panther pathway ontology analysis of both upregulated (**A**) and downregulated (**B**) genes. (**C**) Gene Ontology enrichment analysis using ShinyGO software. Figure shows the fold enrichment of the top 30 identified pathways.

To further understand the results, we performed Gene Ontology using the Panther database (https://pantherdb.org/) by uploading both upregulated and downregulated genes and the results are provided in Figure 7A,B. The upregulated gene list identified total of 62 genes with 92 pathway hits. The top five pathways identified are the angiogenesis signaling pathway, apoptosis signaling pathway, interleukin signaling pathway, integrin signaling pathway, and insulin signaling pathway with genes such as Fos, EGFRI, Itgb6, Irs1, Irs2, HSP1A, etc. (Figure 7A). The downregulated list of genes identified 43 pathway hits with 72 genes passing the criteria. The top five pathway identified by the downregulated list is Beta 1,2,3 adrenergic pathway, metabotropic glutamate receptor pathway III, etc., with key genes identified such as Gnb3, Mmp8, Slc17a7, etc.

We also performed gene set enrichment analysis using ShinyGO 0.77 online software, version 0.77 (http://bioinformatics.sdstate.edu/go77/) with all differentially regulated genes with an FDR cutoff of 0.5, and the results of top 30 hits are shown in Figure 7C. The complete list with enrichment score is provided in Table S2. This analysis identified pathways such as the longevity-regulating pathway (Hspa8 Irs2 Irs1 Hspa1a), IL-17 signaling pathway (Fosb Fos Lcn2 Cxcl10), FoxO signaling pathway (Gadd45b Gadd45g Plk2 Irs2 Irs1), insulin resistance (Tbc1d4 Irs2 Irs1 Ppp1r3c), MAPK signaling pathway (Gadd45b Hspa8 Fos Gadd45g Myc Nr4a1 Hspa1a), etc. The enrichment FDR and fold enrichment scores are provided in Table 7.

**Table 7:** Gene enrichment analysis.

Enrichment FDR	nGenes	Pathway Genes	Fold Enrichment	Pathway	Genes
0.00656708	3	24	20.26388889	Asthma	H2-Aa H2-Eb1 H2-Ab1
3.24 × 10^−7^	9	80	18.2375	Antigen processing and presentation	Hspa8 Cd4 Cd74 H2-Aa H2-Eb1 H2-K1 H2-Q6 H2-Ab1 Hspa1a
0.000640951	5	53	15.29350105	Allograft rejection	H2-Aa H2-Eb1 H2-K1 H2-Q6 H2-Ab1
0.000640951	5	53	15.29350105	Graft-versus-host disease	H2-Aa H2-Eb1 H2-K1 H2-Q6 H2-Ab1
0.001010066	5	60	13.50925926	Type I diabetes mellitus	H2-Aa H2-Eb1 H2-K1 H2-Q6 H2-Ab1
0.014935096	3	37	13.14414414	Thyroid cancer	Gadd45b Gadd45g Myc
0.001739711	5	69	11.74718196	Autoimmune thyroid disease	H2-Aa H2-Eb1 H2-K1 H2-Q6 H2-Ab1
0.018879359	3	42	11.57936508	Intestinal immune network for IgA production	H2-Aa H2-Eb1 H2-Ab1
0.007502182	4	62	10.45878136	Longevity-regulating pathway	Hspa8 Irs2 Irs1 Hspa1a
0.000350761	7	109	10.4108053	Toxoplasmosis	Hspa8 H2-Aa H2-Eb1 Irgm2 H2-Ab1 Igtp Hspa1a
0.00250099	5	78	10.39173789	Viral myocarditis	H2-Aa H2-Eb1 H2-K1 H2-Q6 H2-Ab1
0.003489764	5	86	9.425064599	Rheumatoid arthritis	Fos Ccl5 H2-Aa H2-Eb1 H2-Ab1
0.009996389	4	69	9.397745572	Leishmaniasis	Fos H2-Aa H2-Eb1 H2-Ab1
0.003489764	5	87	9.316730524	Th1 and Th2 cell differentiation	Fos Cd4 H2-Aa H2-Eb1 H2-Ab1
0.010543292	4	71	9.133020344	Drug metabolism	Mgst1 Aldh3b3 Fmo2 Aox1
0.00656708	5	104	7.793803419	Th17 cell differentiation	Fos Cd4 H2-Aa H2-Eb1 H2-Ab1
0.018879359	4	88	7.368686869	Colorectal cancer	Gadd45b Fos Gadd45g Myc
0.019277334	4	91	7.125763126	IL-17 signaling pathway	Fosb Fos Lcn2 Cxcl10
0.019315597	4	92	7.048309179	Hematopoietic cell lineage	Cd4 H2-Aa H2-Eb1 H2-Ab1
0.000254132	10	240	6.75462963	Human T-cell leukemia virus 1 infection	Fos Myc Cd4 H2-Aa Egr1 Zfp36 H2-Eb1 H2-K1 H2-Q6 H2-Ab1
0.001990294	7	169	6.714661407	Influenza A	Dnajb1 Il33 Cxcl10 Ccl5 H2-Aa H2-Eb1 H2-Ab1
0.000476756	9	220	6.631818182	Epstein–Barr virus infection	Gadd45b Gadd45g Myc Cxcl10 H2-Aa H2-Eb1 H2-K1 H2-Q6 H2-Ab1
0.013590203	5	131	6.187446989	FoxO signaling pathway	Gadd45b Gadd45g Plk2 Irs2 Irs1
0.00656708	6	160	6.079166667	Cell adhesion molecules	Cd4 H2-Aa H2-Eb1 H2-K1 H2-Q6 H2-Ab1
0.031943118	4	109	5.9490316	Insulin resistance	Tbc1d4 Irs2 Irs1 Ppp1r3c
0.009005564	6	174	5.590038314	Cellular senescence	Gadd45b Zfp36l1 Gadd45g Myc H2-K1 H2-Q6
0.028314125	5	169	4.796186719	Phagosome	H2-Aa H2-Eb1 H2-K1 H2-Q6 H2-Ab1
0.018879359	6	215	4.524031008	Kaposi sarcoma-associated herpesvirus infection	Fos Myc Gnb3 Zfp36 H2-K1 H2-Q6
0.023019765	6	231	4.210678211	Human immunodeficiency virus 1 infection	Fos Cd4 Gnb3 Trim30d H2-K1 H2-Q6
0.019277334	7	294	3.85978836	MAPK signaling pathway	Gadd45b Hspa8 Fos Gadd45g Myc Nr4a1 Hspa1a

### 5.2. Gel Shift and Western Blot Analysis Shows PHD1^−/−^ MI Mice Had Significantly Increased HIF 1α Expression, HSPA12B, and IRS2 Compared to WTMI Mice

**Figure 8:**
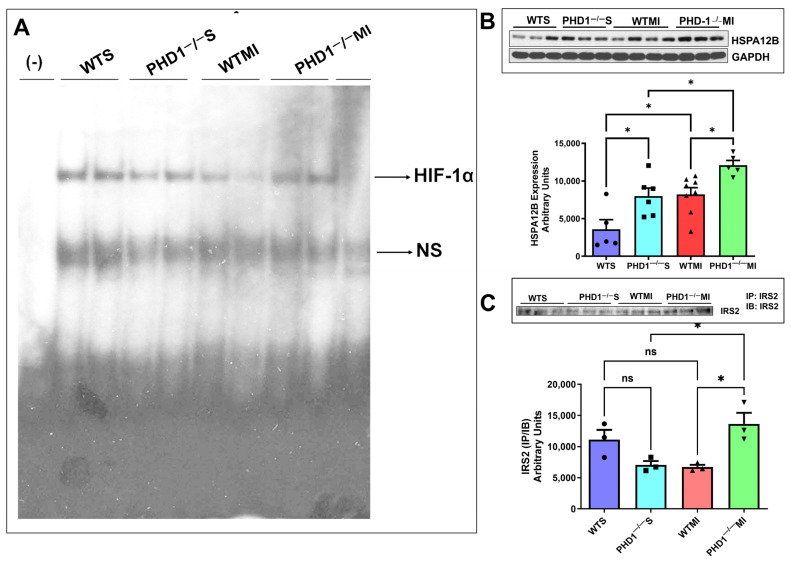
(**A**) Image of gel shift analysis performed on each group of mice, showing a significant reduction in HIF-1α in the WTMI group compared to the PHD1^−/−^ MI group. ns is indicative of non-specific free probes detected in all groups (n = 2). (**B**) The immunoblotting analysis compared all four mice groups, using GAPDH as a loading control. The bar graph shows a significant increase in HSPA12B level in PHD1^−/−^ S compared to the WTS group and PHD1^−/−^ MI group compared to the WTMI group (n = 5–8; values are mean ± SEM (* *p* < 0.05). Asterisks represent the degree of significance between the two groups). (**C**) Immunoblotting analysis was performed comparing Insulin Receptor Substrate-2 IP/IB expression in all four groups of mice. Bar graph showing no significant difference in IRS2 (IP/IB) expression in PHD1^−/−^ S mice when compared to WTS mice, but the PHD1^−/−^ MI group shows a significant increase in the expression of IRS2 (IP/IB) when compared to the WTS group. Values are mean ± SEM (* *p* < 0.05). Asterisks represent the degree of significance between the two groups, ns represents not significant.

Gel shift analysis for HIF1α showed no significant changes in the sham groups. However, the PHD1^−/−^ mice showed increased HIF-1α expression after MI compared to the WTMI group (Figure 8A). The Western blot analysis for the heat shock protein HSPA12B is shown in Figure 8B. The expression level of the protein HSPA12B was increased in the PHD1^−/−^Sham group as compared to the WT sham group (*p* = 0.0283, n = 3). MI group comparisons also showed significantly increased HSPA12B (small-molecular-weight heat shock protein) expression in PHD1^−/−^ mice compared to WT (*p* = 0.0433. n = 3) (Figure 8B). We also selected one of the genes, IRS2, which was increased in Affymetrix analysis and validated by Western blot analysis. We performed immunoprecipitation and immunoblotting of IRS2 protein using IRS2 antibody. Our results showed no significant difference between the sham groups. However, we found significantly increased expression of IRS2 protein in the PHD1^−/−^MI group compared to the WTMI group (*p* = 0.0252, n = 3) (Figure 8C).

## 6. Discussion

Our previous studies have demonstrated the beneficial effects of inhibiting these PHD enzyme (PHD2 and PHD3) isoforms in genetically knockout mice by simulating myocardial infarction with permanent ligation of the left anterior descending artery. With the advent of oral formulation of a novel PHD inhibitor in a phase one clinical study [28], there is a rekindled interest in the PHD knockout pathway to ameliorate the effects of ischemic insult in the setting of myocardial infarction. In this study, we focused on PHD1, one of the three isoforms of the PHD enzymes.

PHD1 knockout mice exhibit a similar upregulation and stabilization of HIF1α (Figure 8) following myocardial infarction as our previous studies with PHD 2 and PHD 3 knockout mice. PHD1 mice were subjected to MI, showing a remarkable preservation of cardiac function after the insult compared to wild-type mice, as evidenced by the superior ejection fraction and cardiac output post-MI. Stabilizing the HIF pathway brings about these effects and subsequent increased levels of HIF-1 expression, as demonstrated in our gel shift analysis, marking a significant advancement in our understanding of cardiac function preservation.

**Figure 9:**
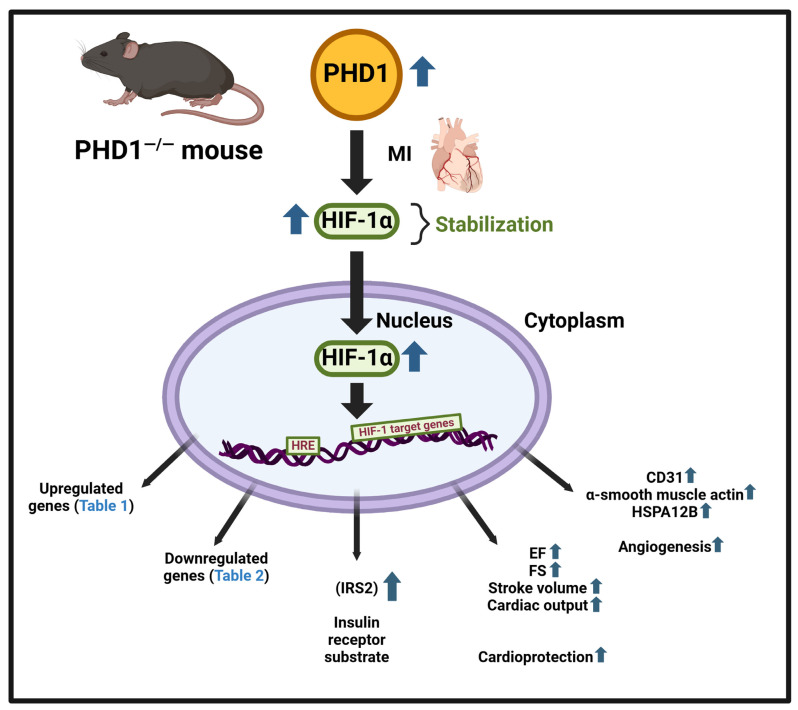
Flow diagram denoting that PHD1 knockout in mouse genetic model leads to increased angiogenesis and cardioprotection. In addition, GeneChip™ analysis designated 174 differentially expressed gene (Table 1 and Table 2). Bio Render software (version 1.0.0.3) was used to create this figure.

On the tissue level, knockout of the PHD1 enzyme resulted in increased capillary and arteriolar density. Using anti-CD31 and anti-α-smooth muscle actin, we illustrated the increase in vessel density at the myocardial risk area, confirming the proangiogenic effect of the knockout of PHD1. This preservation of the arteriolar and capillary network may contribute to the subsequent preservation of cardiac function (Figure 9).

In our present study, our focus was to identify differentially regulated gene expression by the knockout of PHD1 in a mouse MI model. Due to advances in personalized medicine and therapies, there is a growing focus on identifying genes causing diseases for diagnosis and prognosis. In recent years, bioinformatics has played a significant role in omics techniques, particularly in gene array or RNA-sequencing (RNA-seq) technologies to identify vast amounts of data and differentially regulated gene expression levels in control and experimental groups. Our results provide a direction for further studies, with 174 differentially expressed genes identified so far, including 89 upregulated (Table 1) and 85 downregulated (Table 2) genes. A graphical summary of the IPA analysis is provided in Figure 5, which shows several important pathways and genes that have been identified due to PHD1 knockout. The PHD1^−/−^ KO mouse colony was mainly generated to identify specific mechanisms and molecules involved in cardioprotection after myocardial infarction. Pathway enrichment analysis or networks identified from Ingenuity pathway analysis are listed in Table 3, which predicts the function of the differentially expressed genes, including the molecular mechanism, biological process, and cellular components. An in-depth analysis of the genes using the network analysis tool in IPA revealed 13 gene interaction networks. Among these networks, Network 5, Network 11, Network 12, and Network 13 are the most significant and have been found to be linked to cardiovascular disease. Important molecules involved in these network analyses are VEGF, HSP70, NADPH, NOTCH, PDGF, STAT5a/b, Cytochrome C, SOD, PI3K, SMAD2/3, P38MAPK, and many more (Table 3). As shown in Figure 6, these molecules play a significant role in cardiovascular function, development, differentiation, cellular assembly, molecular transport, and cell survivability. We also performed regulator effect analysis in IPA, identifying 22 key regulator molecules such as KLF2, EGF, FGF2, IGF1R, TP63, CXCL12, NGF, VEGFA, YAP1, etc. (see Table 5 for complete list), and their effect on the predicted causes and effects of key protein molecules predicted to regulate the proliferation of vascular smooth muscle cells. The concept of “regulator effects” describes how anticipated upstream regulators that are either activated or inhibited may result in downstream phenotypic or functional consequences that are either increased or decreased. The detailed list of 22 regulator molecules is given in Table 5, along with their target molecules in the dataset. These molecules could be the reasons for increased vessel density and cardioprotective effects in the PHD1^−/−^MI group compared to the WTMI group.

Gene Ontology enrichment analysis using the ShinyGO software platform revealed multiple biological processes which are regulated in the absence of PHD1. All the biological pathways identified independently by GO analysis and canonical pathways by IPA analysis denote a strong correlation between pathways. GO enrichment analysis shows the fold enrichment of 30 identified pathways (Figure 7). Among these pathways, the longevity-regulating pathway, type 1 diabetes, MAPK signaling, and cellular senescence are prominent. Many other biological signaling pathways were also identified in this study with Shiny GO software (version 0.77) analysis; some of them have not been previously characterized. This study identifies at least two downstream potential target proteins, HSPA12B (a survival protein) and IRS2 (a major substrate of insulin receptor), validated at the protein level. These two proteins are found to be increased in the absence of PHD1 in MI settings.

There are very few FDA-approved drugs to adopt this strategy to inhibit PHDs for therapeutic potential. This benefit is currently being utilized in clinical investigations as Roxadustat (FG-4592), a PHD inhibitor [29], has been approved in China, Japan, and Chile and is under review in the US. Roxadustat is a PHD inhibitor that stabilizes HIF accumulation and translocation to the nuclei. There is a new class of FDA-approved PHD inhibitors, GSK1278863, also known as Daprodustat. This is the only drug that is FDA-approved. There are a series of PHD inhibitors, such as Desidustat, Enarodustat, Molidustat and Vadadustat. Various companies manufacture these drugs to treat anemia of chronic kidney disease. Therefore, the present study illuminates the potential for PHD (isoforms) inhibitors for the treatment of affected individuals exposed to ischemia but at the same time highlights the need to identify other side effects due to drug exposure.

In conclusion, our results shed light on the efficacy of PHD1 knockout in activating several important molecules, signaling pathways and networks, resulting in cardioprotection against myocardial infarction, suggesting the need to examine PHD1 for its therapeutic potential further. This results also identifies two important target molecules, IRS2 and HSPA12B, downstream of PHD1, which play a significant role in cellular defense, survivability, and angiogenesis. In addition, this work also suggests a new role of PHD1 in cell differentiation, cellular senescence (aging), and survivability.

## Figures and Tables

**Table 1:** List of selected upregulated genes.

	Gene Symbol	Description	Fold Change	*p*-Value
1	Mtss1	metastasis suppressor 1	1.5	0.0007
2	Dennd4a	DENN/MADD domain containing 4A	1.51	0.0027
3	Abra	actin-binding Rho activating protein	1.51	0.0045
4	Fam13a	family with sequence similarity 13, member A	1.51	0.037
5	Kcnn2	potassium intermediate/small conductance calcium-activated channel, subfamily N, member 2	1.51	0.0459
6	Myc	myelocytomatosis oncogene	1.52	0.014
7	Iigp1; Iigp1b	interferon inducible GTPase 1; interferon inducible GTPase 1B	1.52	0.0299
8	Tbc1d4	TBC1 domain family, member 4	1.53	0.0007
9	Prss55	protease, serine 55	1.53	0.0008
10	Igtp; Irgm2	interferon gamma induced GTPase; immunity-related GTPase family M member 2	1.53	0.0034
11	Aplnr	apelin receptor	1.53	0.0162
12	Ttc28	tetratricopeptide repeat domain 28	1.54	0.0004
13	Ctgf	connective tissue growth factor	1.54	0.0024
14	Rnf125	ring finger protein 125	1.55	0.0418
15	Irs2	insulin receptor substrate 2	1.56	3.57 × 10^−5^
16	Slc9a9	solute carrier family 9 (sodium/hydrogen exchanger), member 9	1.56	0.0048
17	Pde7a	phosphodiesterase 7A	1.56	0.0245
18	Hmcn1	hemicentin 1	1.57	0.0095
19	Dnajb1	DnaJ (Hsp40) homolog, subfamily B, member 1	1.57	0.0115
20	Pydc4	pyrin domain containing 4	1.57	0.0405
21	Egr1	early growth response 1	1.58	0.0037
22	H2-Eb1	histocompatibility 2, class II antigen E beta	1.58	0.004
23	Rasgef1b	RasGEF domain family, member 1B	1.58	0.0312
24	mt-Tr	mitochondrially encoded tRNA arginine [Source:MGI Symbol;Acc:MGI:102476]	1.59	0.001
25	1810032O08Rik	RIKEN cDNA 1810032O08 gene	1.6	0.0002
26	Cd4	CD4 antigen	1.6	0.0018
27	Gadd45g	growth arrest and DNA-damage-inducible 45 gamma	1.6	0.018
28	Dennd4a	DENN/MADD domain containing 4A	1.6	0.0198
29	Sprr2e	small proline-rich protein 2E	1.61	0.0186
30	Zfp36	zinc finger protein 36	1.62	0.0002
31	Hmcn1	hemicentin 1	1.62	0.0268
32	Airn	antisense Igf2r RNA	1.63	0.0001
33	Apold1	apolipoprotein L domain containing 1	1.63	0.0226
34	Ifi44	interferon-induced protein 44	1.63	0.0289
35	Zfp36l1	zinc finger protein 36, C3H type-like 1	1.64	0.0005
36	Irs1	insulin receptor substrate 1	1.64	0.0023
37	Gm11709	predicted gene 11709	1.64	0.0029
38	Airn	antisense Igf2r RNA	1.64	0.0041
39	Snord14d; Snord14c; Hspa8	small nucleolar RNA, C/D box 14D; small nucleolar RNA, C/D box 14C; heat shock protein 8	1.65	0.0042
40	Cd74	CD74 antigen (invariant polypeptide of major histocompatibility complex, class II antigen-associated)	1.65	0.018
41	Hmcn1	hemicentin 1	1.65	0.0272
42	Rasl11b	RAS-like, family 11, member B	1.68	3.25 × 10^−5^
43	Mamstr	MEF2 activating motif and SAP domain containing transcriptional regulator	1.68	0.0201
44	Gpr65	G-protein coupled receptor 65	1.69	0.0056
45	Bmp4	bone morphogenetic protein 4	1.69	0.0092
46	Nr1d1	nuclear receptor subfamily 1, group D, member 1	1.69	0.0475
47	Gatm	glycine amidinotransferase (L-arginine:glycine amidinotransferase)	1.7	0.0001
48	Myh7b	myosin, heavy chain 7B, cardiac muscle, beta	1.7	0.0004
49	Cnksr3	Cnksr family member 3	1.7	0.0006
50	Gm24564	predicted gene, 24564 [Source:MGI Symbol;Acc:MGI:5454341]	1.72	0.003
51	Dip2c	DIP2 disco-interacting protein 2 homolog C (Drosophila)	1.72	0.026
52	Hspa1a	heat shock protein 1A	1.72	0.0367
53	Gadd45b	growth arrest and DNA-damage-inducible 45 beta	1.73	0.0005
54	Ackr3	atypical chemokine receptor 3	1.74	0.0022
55	Sema4c	sema domain, immunoglobulin domain (Ig), transmembrane domain (TM) and short cytoplasmic domain, (semaphorin) 4C	1.74	0.0067
56	Ifit1	interferon-induced protein with tetratricopeptide repeats 1	1.74	0.0108
57	Gm11710; Cd300lh	predicted gene 11710; CD300 antigen like family member H	1.76	0.0011
58	Snord16a	small nucleolar RNA, C/D box 16A	1.76	0.0178
59	Nr4a1	nuclear receptor subfamily 4, group A, member 1	1.77	0.0043
60	Hmcn1	hemicentin 1	1.78	0.0416
61	Cyr61	cysteine rich protein 61	1.8	0.0006
62	Ccl5	chemokine (C-C motif) ligand 5	1.84	2.82 × 10^−5^
63	Gm11711	predicted gene 11711	1.84	0.001
64	Fos	FBJ osteosarcoma oncogene	1.84	0.0018
65	Plk2	polo-like kinase 2	1.86	0.001
66	Gm4841	predicted gene 4841	1.87	0.0011
67	Fosb	FBJ osteosarcoma oncogene B	1.87	0.0089
68	Gm20481	predicted gene 20481 [Source:MGI Symbol;Acc:MGI:5141946]	1.87	0.0467
69	H2-K1	histocompatibility 2, K1, K region	1.89	0.0119
70	Itgb6	integrin beta 6	1.94	0.0048
71	Cxcl10	chemokine (C-X-C motif) ligand 10	1.94	0.0087
72	LOC105247125	uncharacterized LOC105247125	1.96	0.0017
73	Bach2	BTB and CNC homology 2	1.96	0.0047
74	Atf3	activating transcription factor 3	1.99	0.001
75	H2-Aa	histocompatibility 2, class II antigen A, alpha	2	0.0115
76	H2-Ab1	histocompatibility 2, class II antigen A, beta 1	2.18	0.0025
77	Otud1	OTU domain containing 1	2.53	8.04 × 10^−5^
78	Gdpd3	glycerophosphodiester phosphodiesterase domain containing 3	6.51	0.0136

**Table 2:** List of selected downregulated genes.

	Gene Symbol	Description	Fold Change	*p*-Value
1	Hrasls	HRAS-like suppressor	−4.67	1.44 × 10^−6^
2	Trim12a	tripartite motif-containing 12A	−3.72	0.0004
3	Ppp1r3c	protein phosphatase 1, regulatory (inhibitor) subunit 3C	−2.84	0.0039
4	AA467197; Mir147	expressed sequence AA467197; microRNA 147	−2.73	0.0002
5	Cpxm2	carboxypeptidase X 2 (M14 family)	−2.61	6.47 × 10^−5^
6	C7	complement component 7	−2.23	1.52 × 10^−5^
7	Syt12	synaptotagmin XII	−2.16	0.0154
8	Mmp8	matrix metallopeptidase 8	−2.05	0.0492
9	Entpd4; Gm21685	ectonucleoside triphosphate diphosphohydrolase 4; predicted gene, 21685	−2.04	1.35 × 10^−6^
10	Aldob	aldolase B, fructose-bisphosphate	−2.03	3.35 × 10^−5^
11	Serpina3n	serine (or cysteine) peptidase inhibitor, clade A, member 3N	−2.01	0.0214
12	Clec10a	C-type lectin domain family 10, member A	−2	0.002
13	Prg4	proteoglycan 4 (megakaryocyte-stimulating factor, articular superficial zone protein)	−1.91	0.0237
14	Lrg1	leucine-rich alpha-2-glycoprotein 1	−1.9	0.0019
15	Ngp	neutrophilic granule protein	−1.9	0.0133
16	Serpina3c	serine (or cysteine) peptidase inhibitor, clade A, member 3C	−1.89	0.0033
17	Scn4b	sodium channel, type IV, beta	−1.89	0.0045
18	Slc17a7	solute carrier family 17 (sodium-dependent inorganic phosphate cotransporter), member 7	−1.88	5.44 × 10^−6^
19	Il33	interleukin 33	−1.87	0.0309
20	Bdh1	3-hydroxybutyrate dehydrogenase, type 1	−1.84	0.0003
21	Vsig4	V-set and immunoglobulin domain containing 4	−1.84	0.0009
22	Ahsg	alpha-2-HS-glycoprotein	−1.83	0.0002
23	Nrn1	neuritin 1	−1.83	0.0063
24	Ly6g	lymphocyte antigen 6 complex, locus G	−1.83	0.0242
25	Zfp697	zinc finger protein 697	−1.82	0.0179
26	Cd300ld	CD300 molecule-like family member d	−1.79	0.0018
27	Ptgds	prostaglandin D2 synthase (brain)	−1.78	0.0001
28	Mgst1	microsomal glutathione S-transferase 1	−1.75	0.0087
29	Aox1	aldehyde oxidase 1	−1.73	0.0004
30	Scarb2	scavenger receptor class B, member 2	−1.72	3.49 × 10^−6^
31	Abat	4-aminobutyrate aminotransferase	−1.72	8.88 × 10^−6^
32	Lcn2	lipocalin 2	−1.71	0.0191
33	Olfr1418	olfactory receptor 1418	−1.71	0.0208
34	Osr1	odd-skipped related 1 (Drosophila)	−1.7	9.46 × 10^−5^
35	Casc4	cancer susceptibility candidate 4	−1.7	0.0002
36	Lcmt2	leucine carboxyl methyltransferase 2	−1.7	0.0004
37	Slco2b1	solute carrier organic anion transporter family, member 2b1	−1.7	0.0063
38	Cyp1b1	cytochrome P450, family 1, subfamily b, polypeptide 1	−1.69	4.16 × 10^−5^
39	Apod	apolipoprotein D	−1.69	0.0002
40	Snora21	small nucleolar RNA, H/ACA box 21	−1.69	0.02
43	Arg1	arginase, liver	−1.68	0.0215
41	Pgap2	post-GPI attachment to proteins 2	−1.67	3.44 × 10^−6^
42	Fmo2	flavin containing monooxygenase 2	−1.67	0.0019
43	Gm7697	predicted gene 7697	−1.66	0.0011
44	Gm20946	predicted gene, 20946 [Source:MGI Symbol;Acc:MGI:5434301]	−1.64	0.0004
45	Hand2	heart and neural crest derivatives expressed transcript 2	−1.64	0.0005
46	Lnx1	ligand of numb-protein X 1	−1.64	0.0012
47	Gm9495	predicted gene 9495	−1.63	0.0004
49	Gcnt1	glucosaminyl (N-acetyl) transferase 1, core 2	−1.63	0.0302
50	Ano5	anoctamin 5	−1.62	0.0004
51	Ankrd9	ankyrin repeat domain 9	−1.6	0.0011
52	Ucp3	uncoupling protein 3 (mitochondrial, proton carrier)	−1.59	0.0002
53	Gnb3	guanine nucleotide binding protein (G protein), beta 3	−1.59	0.0003
54	Usp17ld	ubiquitin specific peptidase 17-like D	−1.59	0.0004
55	Gm7265	predicted gene 7265	−1.59	0.0152
56	Amy1	amylase 1, salivary	−1.58	0.0009
57	Gpr22	G protein-coupled receptor 22	−1.58	0.016
58	Ttll7	tubulin tyrosine ligase-like family, member 7	−1.57	0.0006
59	Mal	myelin and lymphocyte protein, T cell differentiation protein	−1.56	4.18 × 10^−5^
60	Rasl10b	RAS-like, family 10, member B	−1.56	0.0001
61	Gm7120	predicted gene 7120	−1.56	0.0004
62	Trim30d	tripartite motif-containing 30D	−1.56	0.0012
63	Idh1	isocitrate dehydrogenase 1 (NADP+), soluble	−1.56	0.003
64	Ednra	endothelin receptor type A	−1.56	0.0243
65	Aldh3b3	aldehyde dehydrogenase 3 family, member B3	−1.56	0.0281
66	Serpina3m	serine (or cysteine) peptidase inhibitor, clade A, member 3M	−1.55	0.0025
67	Phka1	phosphorylase kinase alpha 1	−1.54	0.001
68	Pfkfb1	6-phosphofructo-2-kinase/fructose-2,6-biphosphatase 1	−1.54	0.0062
69	Abhd6	abhydrolase domain containing 6	−1.53	0.0043
70	Mfap5	microfibrillar associated protein 5	−1.53	0.0163
71	Chp1	calcineurin-like EF hand protein 1	−1.52	5.06 × 10^−6^
72	Scn5a	sodium channel, voltage-gated, type V, alpha	−1.51	4.44 × 10^−7^
73	Mrap	melanocortin 2 receptor accessory protein	−1.51	0.0034

**Table 3:** List of networks identified from ingenuity pathway analysis.

Network	Molecules in Network	Score	Focus Molecules	Top Diseases and Functions
1	ABRA, Akt, BDH1, Calcineurin (complex), Calcineurin A, calpain, CaMKII, Cdk (family), CHP1, Cyclin A, Cyclin D, E2f, Fam13a, FcER1, Gsk3, HAND2, Igtp, MAC, MEF2, MFAP5, MTSS1, NADPH oxidase, Nedd4, NFAT (complex), Ngp, Osteocalcin, PDGF (complex), PFKFB1, PHKA1, PLA2, RASL11B, Rb, SCN4B, SCN5A, STAT3/STAT3	29	15	[Hereditary Disorder, Molecular Transport, Neurological Disease]
2	ABAT, ADCY, ALDOB, Alp, AMY2A, ANKRD9, APOD, ATF3, Calmodulin, CK2, Collagen Alpha1, Creb, EGLN, FOSB, GDPD3, GNRH, IgG, Insulin, IRS1, IRS2, Jnk, Kcnn2, LCN, LCN2, Myosin, Nos, NR4A1, NRN1, Pde, PKA, PRKD, Proinsulin, PTGDS, ROCK, Rsk	29	15	[Cell Morphology, Digestive System Development and Function, Endocrine System Development and Function]
3	2-palmitoylglycerol, ABHD6, Aldh3b3, CCL1, cyclic AMP, DIP2C, DYRK3, FASTK, FENDRR, GATM, GPR65, HEBP1, IFI44, IFIT1B, Iigp1, IL6, Interferon-α Induced, miR-511-5p (miRNAs w/seed UGUCUUU), monooleylphosphatidic acid, MRAP, NPS, PGLYRP2, PLAAT1, Prg4, RASGEF1B, SLC9A9, STAT5B, TGFB1, TLR4, TP53, TRIL, Trim12a, TRIM67, Wfdc17, ZNF697	29	15	[Cellular Development, Connective Tissue Development and Function, Tissue Development]
4	ABHD18, AMPK, APOLD1, caspase, CD3, CG, CNKSR3, cytochrome C, cytochrome-c oxidase, EGR1, ERK, GADD45B, GATM, HMCN1, Hsp90 (family), IL1, IL33, JINK1/2, LH, MAP2K1/2,Mek, Notch, p70 S6k, PARP, PP2A, PRKAA, Raf, SEMA4C, TCR, TH2 Cytokine, TTC28, VSIG4, ZFP36, ZFP36L1	24	13	[Cellular Function and Maintenance, Molecular Transport, RNA Damage and Repair]
5	26S proteasome, Adaptor protein 1, Ap1, ARG1, BMP4, CCL5, CYP1B1, F Actin, Ficolin-rich granule lumen proteins, FSH, Histone h2a, Histone h3, Histone h4, HLA-A, HSPA1A/HSPA1B, HSPA8, IDH1, IFI16, IFN gamma, IKK (complex), LCMT2, LNX1, MHC CLASS I (family), MTORC1, NFkB (family), P110, p85 (pik3r), PI3K p85, Pkc(s), SCARB2, SERPINA3, STAT5a/b, trypsin, Ubiquitin, Vegf	24	13	[Cell Death and Survival, Cellular Compromise, Embryonic Development]
6	ANO5, AOX1, Ap1 gamma, APP, ARNT2, Atp5f1e, BC023105, CD300LB, Cd52, CDK5R2, CPLX1, CPXM2, EGFR, ENTPD4, Gm3893, Gm4841, GNRH2, GOLM2, GPR6, HDL/cholesterol, hexanoic acid, Iigp1, IRF2BP2, MAPT, MGAT4A, NXPE3, peptidase, PLVAP, RASL10B, SERPINF2, SLFN13, Synaptotagmin, SYT12, TMEM267, TOR4A	22	12	[Cellular Assembly and Organization, Neurological Disease, Organismal Injury and Abnormalities]
7	ADRB, AHSG, Airn, CD300LD, CLEC10A, CXCL10, HDL, HISTONE, Hsp27, Ifn, IFN alpha/beta, IFN Beta, IFN type 1, Ifnar, IL12 (complex), JAK, LDL, MAL, NFkB (complex), NfkB-RelA, NfkB1-RelA, Nr1h, Plk, PLK2, pro-inflammatory cytokine, PRSS55, SAA, secretory granule lumen proteins, Serine Protease, Sod, TBC1D4, Tlr, Tnf (family), Trim30a/Trim30d, Usp17la (includes others)	20	11	[Cell Death and Survival, Cellular Function and Maintenance, Hematological System Development and Function]
8	alpha beta dimer:Ii trimer:calnexin, BACH2, BCR (complex), C15orf48, C7,CD74/MHC2A/MHC2B, Clathrin coated MHC class II alpha/beta/Ii nonamer, Clathrin coated MHC class II alpha/beta/Ii nonamer b, FOS, GCNT1, hemoglobin, HLA-DQB1, IgA, IgG1, IgG2a, IgG2b, IgG3, IgM, IKKA/B, Immunoglobulin, LAG3:MHC II, Ly6a (includes others), MGST1, MHC class II alpha/beta/Ii nonamer, MHC II alpha beta heterodimer, MHC II-β, Mhc2 Alpha, MHC2A/MHC2B/peptide fragment, Nck, PDE7A, peptide free MHC II:HLA-DM, PI3K (family), SLCO2B1, Smad2/3, Sos	17	10	[Cell Death and Survival, Connective Tissue Disorders, Gastrointestinal Disease]
9	Activated ZAP-70, Antigen-bearing MHC Class II: TCR complex:CD4:Lck(Y505), AP-1 Clathrin-coated nonameric, AP-1 Complex:Arf1-GTP:Clathrin Triskelion:Nonameric, CD4, CD74, collagen type i (family), cytokine, GADD45G, HLA-DQA1, HLA-DRB5, IFNG-regulated genes with GAS promoter elements, IgE, IL12 (family), Interferon alpha, Mapk, MERTK, MHC, MHC class I (complex), MHC class II (complex), MHC II, MHC/antigen, Nfat (family), Ngf, Nonameric complex in COPII vesicle, P38 MAPK, PI(4)P:AP-2:clathrin:ITSNs:EPS15:REPS1:SGIP1:NECAPs:AAK1:CLASP proteins: cargo: F-BAR proteins: BAR domain proteins: ARP2/3 complex: WASL: f-actin: HIP dimers: DNM: GDP: SYNJs: auxilin: HSPA8: ATP, PLC gamma, PPP1R3C, RNA polymerase II, Rnf125, Tgf beta, TGN-lysosome vesicle with nonameric, TTLL7, ZAP-70 and ITK tyrosine kinases	15	9	[Endocrine System Disorders, Gastrointestinal Disease, Immunological Disease]
10	Ap2, Cbp/p300, DENND4A, DNAJB1, DNAJB1/HSP70, Dynamin, Fascin, Hdac, heat shock-inducible proteins, histone deacetylase, HSP, HSP40:HSP70:ADP:nascent protein, HSP40:HSP70:ATP:nascent protein, Hsp70, HSP70 co-chaperone, HSP70: DNAJB1, HSP70: DNAJB1:Ac-K80-HSF1, HSP70:DNAJB1:Ac-K80-HSF1 trimer:target gene, HTT aggregate, MYC, MYH7B, N-CoR, OSR1, PFK, PP1, Rar, SERCA, SIRT1:HSP70:DNAJB1:Ac-K80-HSF1:target gene, SMAD1/5/9, Sprr2e, STAT1 Dimer, thyroid hormone receptor, Tropomyosin, UCP3, YAP/TAZ	11	7	[Molecular Transport, Nucleic Acid Metabolism, Small Molecule Biochemistry]
11	Activin (family), CCN1, CCN2, collagen, Collagen type I (complex), Collagen type II, Collagen type IV, Collagen(s), collagenase, elastase, ERK1/2, FAM20C:FAM20C substrates, FGFR, gelatinase, GGTase I, growth hormone, HLA/DQ, IGF receptor, Integrin, ITGB6, Laminin (complex), LATS, LRG1, Mir122a, b, Mmp, MMP8, OTUD1, PDGF-BB, Pkg, SMAD, specific granule lumen proteins, TCF, TEAD, tertiary granule lumen proteins, TSPAN4	11	7	[Cellular Assembly and Organization, Cellular Movement, Hematological System Development and Function]
12	ACKR3, APLNR, Beta Arrestin, chemokine, EDNRA, Endothelin, estrogen receptor, Focal adhesion kinase, G protein, G protein alpha i, G protein beta gamma core, GNB3, Gpcr, GPR22, GPR65, JAK1/2, Ligand:GPCR complexes that activate Gi, Ligand:GPCR complexes that activate Gi:Heterotrimeric G-protein Gi (active), Ligand:GPCR complexes that activate Gi:Heterotrimeric G-protein Gi (inactive), Ligand:GPCR complexes that activate Gq/11:Heterotrimeric G-protein Gq (active), Ligand:GPCR complexes that activate Gq/11:Heterotrimeric G-protein Gq (inactive), NMDA receptor, P glycoprotein, PI3K (complex), PLC, Rac, RAS, Ras homolog, Sapk, Secretase gamma, Shc, SLC17A7, SRC (family), STAT, TSH	11	7	[Cardiovascular Disease, Congenital Heart Anomaly, Developmental Disorder]
13	4632427E13Rik, AGRP, BCL9, beta-estradiol, CCR3, Cxcl3, CYTH3, DIO1, DIO2, EGR4, FMO1, FMO2, GABRP, GADD45G, GATA2, GNRHR, GPRC6A, GPX3, GREM1, ITGAE, KCND3, LUM, LYL1, Mcpt1, MGST1, MMRN1, N(G)-monomethyl-D-arginine, PLK2, Potefam3e (includes others), Snord16a, Sprr1b, TMPRSS3, ZFHX4, ZNF503, ZNF804A	11	7	[Connective Tissue Development and Function, Embryonic Development, Organismal Development]

**Table 4:** Disease and biofunction cardiac fibrosis.

Categories	Diseases or Functions Annotation	*p*-Value	Molecules	# Molecules
Cardiac Fibrosis	Fibrosis of heart	3.82 × 10^−5^	Airn, ARG1, ATF3, CCN2, CYP1B1, EGR1, HAND2, LCN2, PFKFB1, SCN5A	10
Cardiac Fibrosis	Fibrosis of ventricular wall	0.0217	SCN5A	1
Cardiac Fibrosis	Fibrosis of heart ventricle	0.0306	LCN2, SCN5A	2
Cardiac Fibrosis	Perivascular fibrosis of artery	0.0376	OTUD1	1
Cardiac Dysfunction, Cardiac Fibrosis	Interstitial fibrosis of left ventricle	0.0989	LCN2	1
Cardiac Fibrosis	Myocardial fibrosis	0.156	HAND2	1

**Table 5:** Regulatory molecules and predicted targets identified in proliferation of vascular smooth muscle cells.

ID	Regulators	Target Total	Target Molecules in Dataset
1	KLF2	4	BMP4, CYP1B1, MYC, PTGDS
2	EGF	5	CYP1B1, EGR1, IRS1, MYC, NR4A1
3	FGF2	5	BMP4, EGR1, IRS1, MYC, NR4A1
4	IGF1R	4	EGR1, IRS1, MYC, NR4A1
5	TP63	4	ARG1, BMP4, EGR1, MYC
6	CXCL12	3	EGR1, MYC, NR4A1
7	GROWTH HORMONE (family)	3	BMP4, EGR1, MYC
8	HDAC (family)	3	EGR1, MYC, NR4A1
9	LH (complex)	3	EGR1, MYC, NR4A1
10	MEK (family)	3	EGR1, MYC, NR4A1
11	NGF	3	EGR1, MYC, NR4A1
12	NRG1	3	EGR1, MYC, NR4A1
13	VEGFA	3	BMP4, EGR1, NR4A1
14	YAP1	3	ARG1, EGR1, MYC
15	AGT	7	ARG1, EGR1, IRS1, MMP8, MYC, NR4A1, PTGDS
16	IMMUNOGLOBULIN (complex)	6	ARG1, BMP4, CYP1B1, EGR1, MYC, NR4A1
17	RELA	5	EGR1, MMP8, MYC, NR4A1, PTGDS
18	NFKB1	4	ARG1, EGR1, MYC, NR4A1
19	PDGF-BB (complex)	4	BMP4, EGR1, MYC, NR4A1
20	SOCS3	4	ARG1, EGR1, IRS1, MYC
21	SFTPA1	3	CYP1B1, EGR1, MYC
22	IGF1	6	BMP4, CYP1B1, EGR1, IRS1, MYC, NR4A1

**Table 6:** Selected inflammation mediated affected molecules from ingenuity pathway analysis.

Category	*p*-Value	Molecules
Inflammatory Response	7.41 × 10^−15^–3.57 × 10^−3^	ABAT, ABHD6, ACKR3, AHSG, AMY2A, APOD, ARG1, ATF3, BACH2, BMP4, CCL5, CCN1, CCN2, CD300LD, CD4, CD74, CHP1, CLEC10A, CXCL10, CYP1B1, DIP2C, EDNRA, EGR1, ENTPD4, Fam13a, FOS, FOSB, GADD45B, GADD45G, GATM, GCNT1, GPR65, HLA-A, HLA-DQA1, HLA-DQB1, HLA-DRB5, HSPA1A/HSPA1B, HSPA8, IDH1, IFI16, IFI44, IFIT1B, Igtp, Iigp1, IL33, IRS1, IRS2, ITGB6, LCN2, LRG1, Ly6a (includes others), MERTK, MFAP5, MGST1, MMP8, MYC, Nedd4, NR4A1, OTUD1, PDE7A, PTGDS, SCARB2, SCN4B, SCN5A, SERPINA3, SLC9A9, SLCO2B1, TTC28, VSIG4, ZFP36
Inflammatory Disease	3.3 × 10^−12^–3.46 × 10^−3^	ABAT, ABHD6, ACKR3, AHSG, AMY2A, APOD, ARG1, ATF3, BACH2, BMP4, CCL5, CCN1, CCN2, CD4, CD74, CHP1, CXCL10, CYP1B1, DIP2C, EDNRA, EGR1, ENTPD4, FOS, FOSB, GADD45B, GADD45G, GATM, GPR65, HLA-A, HLA-DQA1, HLA-DQB1, HLA-DRB5, HSPA1A/HSPA1B, HSPA8, IFI16, IFI44, Igtp, Iigp1, IL33, IRS1, IRS2, ITGB6, LCN2, LRG1, Ly6a (includes others), MERTK, MMP8, MYC, NR4A1, OTUD1, PDE7A, PTGDS, SCN4B, SCN5A, SERPINA3, SLCO2B1, TTC28, VSIG4, ZFP36

## Data Availability

The data presented in this study are available on request from the corresponding author.

## Supplementary Materials

The following supporting information can be downloaded at: https://www.mdpi.com/article/10.3390/cells14060423/s1, Table S1: List of Canonical Pathways and the molecules; Table S2: Cardiac Function in Prolyl-4-Hydroxylase 1^−/−^ Mice at 30 Days Post-MI Surgery.
