# Supplementary material for: Stabilization of Transcription Factor, HIF-1α by Prolylhydroxylase 1 Knockout Reduces Cardiac Injury After Myocardial Infarction in Mice
Source: Cells. 2025 Mar 13;14(6):423. doi: 10.3390/cells14060423 (PMC11941588; doi:10.3390/cells14060423)
Supplement: Supplementary file 1 [file cells-14-00423-s001.zip › cells-3377702-supplementary.pdf]

cells MDPI

Table S1. List of Canonical Pathways and the molecules	
Ingenuity Canonical Pathways	Molecules
Macrophage Alternative Activation Signaling Pathway	ARG1,CLEC10A,FOS,HLA-DQA1,HLA-DQB1,HLA-DRB5,IL33,IRS1,IRS2,MYC
ABRA Signaling Pathway	ABRA,CCN2,CHP1,EGR1,FOS,FOSB,HAND2
Antigen Presentation Pathway	CD74,HLA-A,HLA-DQA1,HLA-DQB1,HLA-DRB5
Macrophage Classical Activation Signaling Pathway	CCL5,CXCL10,HLA-DQA1,HLA-DQB1,HLA-DRB5,IL33,MERTK
Th2 Pathway	CD4,HLA-A,HLA-DQA1,HLA-DQB1,HLA-DRB5,IL33
IL-10 Signaling	FOS,HLA-A,HLA-DQA1,HLA-DQB1,HLA-DRB5,IL33
IGF-1 Signaling	CCN1,CCN2,FOS,IRS1,IRS2
Neutrophil degranulation	AHSG,ARG1,HSPA1A/HSPA1B,HSPA8,IDH1,LCN2,LRG1,MGST1,MMP8,SERPINA3
Neuroinflammation Signaling Pathway	CCL5,CHP1,CXCL10,FOS,HLA-A,HLA-DQA1,HLA-DQB1,HLA-DRB5
Th1 and Th2 Activation Pathway	CD4,HLA-A,HLA-DQA1,HLA-DQB1,HLA-DRB5,IL33
IL-17A Signaling in Gastric Cells	CCL5,CXCL10,FOS
MSP-RON Signaling in Macrophages Pathway	ARG1,FOS,HLA-DQA1,HLA-DQB1,HLA-DRB5
Interferon gamma signaling	HLA-A,HLA-DQA1,HLA-DQB1,HLA-DRB5
Interleukin-4 and Interleukin-13 signaling	FOS,HSPA8,LCN2,MYC
MHC class II antigen presentation	CD74,HLA-DQA1,HLA-DQB1,HLA-DRB5
Immunogenic Cell Death Signaling Pathway	CXCL10,HSPA1A/HSPA1B,HSPA8
IL-9 Signaling	IRS1,IRS2
CD28 Signaling in T Helper Cells	CD4,CHP1,FOS,HLA-A,HLA-DQA1,HLA-DQB1,HLA-DRB5
Pulmonary Healing Signaling Pathway	ACKR3,BMP4,MMP8,MYC
PI3K Cascade	IRS1,IRS2
MIF Regulation of Innate Immunity	CD74,FOS
Senescence Pathway	ATF3,CHP1,GADD45B,GADD45G,ZFP36L1
Interleukin-10 signaling	CCL5,CXCL10
CDK5 Signaling	EGR1,FOSB,PPP1R3C
tRNA Splicing	GDPD3,PDE7A
ESR-mediated signaling	FOS,FOSB,MYC
Formation of lateral plate mesoderm	BMP4
Arginine Degradation I (Arginase Pathway)	ARG1
Glutamate Degradation III (via 4-aminobutyrate)	ABAT
Role of Tissue Factor in Cancer	CCN1,CCN2,EGR1,FOS
Sensory perception of taste	GNB3,SCN4B
Clathrin-mediated Endocytosis Signaling	APOD,CHP1,HSPA8,ITGB6

HIF1α Signaling	CHP1,HSPA1A/HSPA1B,HSPA8,MMP8
Apelin Muscle Signaling Pathway	APLNR,GNB3
Neuroprotective Role of THOP1 in Alzheimer's Disease	HLA-A,PRSS55,SERPINA3
LXR/RXR Activation	AHSG,APOD,IL33
ERK/MAPK Signaling	FOS,ITGB6,MYC,PPP1R3C
Regulation of Insulin-like Growth Factor (IGF) transport and uptake by IGFBPs	AHSG,BMP4,CCN1
L1CAM interactions	HSPA8,SCN4B,SCN5A
Activin Inhibin Signaling Pathway	CCN2,FOS,FOSB,IL33
Fructose metabolism	ALDOB
Creatine metabolism	GATM
PKCθ Signaling in T Lymphocytes	CD4,CHP1,FOS,HLA-A,HLA-DQA1,HLA-DQB1,HLA-DRB5
Myelination Signaling Pathway	BMP4,CHP1,FOS,IRS1,ITGB6
Clathrin-mediated endocytosis	CD4,HSPA8,SCARB2
NAFLD Signaling Pathway	FOS,IL33,IRS1,IRS2
Role of Macrophages, Fibroblasts and Endothelial Cells in Rheumatoid Arthritis	CCL5,CHP1,FOS,IL33,MYC
Atherosclerosis Signaling	APOD,IL33,PLAAT1
P2Y Purigenic Receptor Signaling Pathway	FOS,GNB3,MYC
Phagosome Formation	ACKR3,APLNR,EDNRA,GPR22,GPR65,ITGB6,MYH7B,PLAAT1
Atorvastatin ADME	SLCO2B1
Urea Cycle	ARG1
Arginine Degradation VI (Arginase 2 Pathway)	ARG1
Phosphatidylcholine Biosynthesis I	PHKA1
CSDE1 Signaling Pathway	FOS,MYC
SNARE Signaling Pathway	HSPA8,MYH7B,SYT12
NRF2-mediated Oxidative Stress Response	AOX1,DNAJB1,FOS,MGST1
Insulin Receptor Signaling	IRS1,IRS2,PPP1R3C
Apelin Endothelial Signaling Pathway	APLNR,FOS,GNB3
Nicotine Degradation III	AOX1,CYP1B1
AMPK Signaling	GNB3,IRS1,IRS2,PFKFB1
Breast Cancer Regulation by Stathmin1	ACKR3,APLNR,EDNRA,GNB3,GPR22,GPR65,PPP1R3C
Urea cycle	ARG1
Lipophagy	HSPA8
Sucrose Degradation V (Mammalian)	ALDOB
Endocannabinoid Neuronal Synapse Pathway	ABHD6,CHP1,GNB3
Wound Healing Signaling Pathway	CCL5,FOS,IL33,MMP8

Glutamate Receptor Signaling	GNB3,SLC17A7
Factors Promoting Cardiogenesis in Vertebrates	BMP4,MYC,SCN5A
Cachexia Signaling Pathway	HSPA1A/HSPA1B,HSPA8,IL33,IRS1,LCN2
T Cell Receptor Signaling	CD4,CHP1,FOS,HLA-A,HLA-DQA1,HLA-DQB1,HLA-DRB5
Ketone body metabolism	BDH1
Prostanoid Biosynthesis	PTGDS
Citrulline Biosynthesis	ARG1
Ketolysis	BDH1
Role of JAK1 and JAK3 in γc Cytokine Signaling	IRS1,IRS2
Cardiac Hypertrophy Signaling	CHP1,GNB3,HAND2,IRS1
Interferon alpha/beta signaling	EGR1,HLA-A
Growth Hormone Signaling	FOS,IRS1
HEY1 Signaling Pathway	BMP4,MFAP5,MMP8
Ketogenesis	BDH1
Chaperone Mediated Autophagy Signaling Pathway	CHP1,DNAJB1,HSPA1A/HSPA1B,HSPA8,IDH1,MMP8,MYC
ICOS-ICOSL Signaling in T Helper Cells	CD4,CHP1,HLA-A,HLA-DQA1,HLA-DQB1,HLA-DRB5
Extra-nuclear estrogen signaling	FOS,GNB3
Axonal Guidance Signaling	BMP4,CHP1,GNB3,ITGB6,MMP8,SEMA4C
Colorectal Cancer Metastasis Signaling	FOS,GNB3,MMP8,MYC
ERK5 Signaling	FOS,MYC
NUR77 Signaling in T Lymphocytes	CHP1,HLA-A,HLA-DQA1,HLA-DQB1,HLA-DRB5,NR4A1
Caveolar-mediated Endocytosis Signaling	HLA-A,ITGB6
Opioid Signaling Pathway	FOS,FOSB,GNB3,MYC
VDR/RXR Activation	CCL5,CXCL10
Toll-like Receptor Signaling	FOS,IL33
Aldosterone Signaling in Epithelial Cells	DNAJB1,HSPA1A/HSPA1B,HSPA8
Transcriptional regulation of testis differentiation	PTGDS
IL-3 Signaling	CHP1,FOS
Maturity Onset Diabetes of Young (MODY) Signaling	ALDOB,APOD
G alpha (q) signalling events	EDNRA,GNB3,GPR65
Huntington's Disease Signaling	DNAJB1,GNB3,HSPA1A/HSPA1B,HSPA8
Glucose metabolism	ALDOB,PFKFB1
Erythropoietin Signaling Pathway	FOS,IL33,IRS2
Signaling by NTRK1 (TRKA)	IRS1,IRS2
Chemokine Signaling	CCL5,FOS
Aspirin ADME	SLCO2B1

Protein Kinase A Signaling	CHP1,GDPD3,GNB3,PDE7A,PPP1R3C
Tight Junction Signaling	CNKSR3,FOS,MYH7B
YAP1- and WWTR1 (TAZ)-stimulated gene expression	CCN2
Synthesis of Prostaglandins (PG) and Thromboxanes (TX)	PTGDS
Response of EIF2AK1 (HRI) to heme deficiency	ATF3
Choline Biosynthesis III	PHKA1
IL-33 Signaling Pathway	CCL5,FOS,IL33
IL-17A Signaling in Fibroblasts	FOS,LCN2
PDGF Signaling	FOS,MYC
Xenobiotic Metabolism AHR Signaling Pathway	CYP1B1,MGST1
IL-17 Signaling	FOS,IL33,LCN2
Superpathway of Citrulline Metabolism	ARG1
RAR Activation	FOS,GDPD3,IL33,MMP8,PDE7A
GNRH Signaling	EGR1,FOS,GNB3
Production of Nitric Oxide and Reactive Oxygen Species in Macrophages	APOD,FOS,PPP1R3C
Acute Myeloid Leukemia Signaling	IDH1,MYC
Crosstalk between Dendritic Cells and Natural Killer Cells	HLA-A,HLA-DRB5
RANK Signaling in Osteoclasts	CHP1,FOS
Apelin Adipocyte Signaling Pathway	APLNR,MGST1
Protein methylation	HSPA8
Signaling by NTRK3 (TRKC)	IRS1
Germ layer formation at gastrulation	BMP4
MAPK6/MAPK4 signaling	DNAJB1,MYC
Differential Regulation of Cytokine Production in Macrophages and T Helper Cells by IL-17A and IL-17F	CCL5
Natural Killer Cell Signaling	HLA-A,HSPA1A/HSPA1B,HSPA8
CDC42 Signaling	FOS,HLA-A,HLA-DQA1,HLA-DQB1,HLA-DRB5,ITGB6
IL-4 Signaling	ARG1,HLA-DQA1,HLA-DQB1,HLA-DRB5,IRS1,IRS2
Transcriptional regulation by RUNX3	CCN2,MYC
IL-1 Signaling	FOS,GNB3
TGF-β Signaling	BMP4,FOS
Graft-versus-Host Disease Signaling	HLA-A,HLA-DQA1,HLA-DQB1,HLA-DRB5,IL33
Metabolism of cofactors	IDH1
CDX Gastrointestinal Cancer Signaling Pathway	BMP4,FOS,IL33
Gustation Pathway	GNB3,SCN4B,SCN5A
p53 Signaling	GADD45B,GADD45G

Glutaminergic Receptor Signaling Pathway (Enhanced)	CHP1,PLAAT1,SCN4B,SCN5A
Valine Degradation I	ABAT
ATM Signaling	GADD45B,GADD45G
Beta-catenin independent WNT signaling	GNB3,MYC
Digestion	AMY2A
Histamine Degradation	Aldh3b3
Fatty Acid α-oxidation	Aldh3b3
Integration of energy metabolism	GNB3,PFKFB1
Transport of inorganic cations/anions and amino acids/oligopeptides	SLC17A7,SLC9A9
Mouse Embryonic Stem Cell Pluripotency	BMP4,MYC
Chaperone Mediated Autophagy	HSPA8
Putrescine Degradation III	Aldh3b3
CREB Signaling in Neurons	ACKR3,APLNR,EDNRA,GNB3,GPR22,GPR65
Cargo recognition for clathrin-mediated endocytosis	CD4,SCARB2
PPAR Signaling	FOS,IL33
FAK Signaling	ACKR3,APLNR,EDNRA,FOS,GPR22,GPR65,IL33,ITGB6,MYC
Metabolism of porphyrins	SLCO2B1
Formation of paraxial mesoderm	BMP4
RHOGDI Signaling	GNB3,ITGB6,MYH7B
OX40 Signaling Pathway	CD4,HLA-A,HLA-DQA1,HLA-DQB1,HLA-DRB5
Other interleukin signaling	CD4
HER-2 Signaling in Breast Cancer	FOS,ITGB6,MYC
Incretin synthesis, secretion, and inactivation	GNB3
Signaling by Erythropoietin	IRS2
Bupropion Degradation	CYP1B1
Ethanol Degradation IV	Aldh3b3
BBSome Signaling Pathway	ACKR3,APLNR,EDNRA,GPR22,GPR65
S100 Family Signaling Pathway	ACKR3,APLNR,EDNRA,FOS,GPR22,GPR65,MMP8
IL-13 Signaling Pathway	ARG1,IL33
Bladder Cancer Signaling	MMP8,MYC
Estrogen-mediated S-phase Entry	MYC
Neurovascular Coupling Signaling Pathway	ENTPD4,Kcnn2,PLAAT1
Neuregulin Signaling	ITGB6,MYC
Virus Entry via Endocytic Pathways	HLA-A,ITGB6
Effects of PIP2 hydrolysis	ABHD6
ATF4 activates genes in response to endoplasmic reticulum stress	ATF3

Cardiomyocyte Differentiation via BMP Receptors	BMP4
Tryptophan Degradation X (Mammalian, via Tryptamine)	Aldh3b3
Glycolysis I	ALDOB
Gluconeogenesis I	ALDOB
Cholecystokinin/Gastrin-mediated Signaling	FOS,IL33
Sertoli Cell-Germ Cell Junction Signaling Pathway (Enhanced)	FOS,FOSB,MMP8
cAMP-mediated signaling	APLNR,GDPD3,PDE7A
p38 MAPK Signaling	IL33,MYC
Systemic Lupus Erythematosus in T Cell Signaling Pathway	CHP1,FOS,HLA-A,HLA-DQA1,HLA-DQB1,HLA-DRB5
Renin-Angiotensin Signaling	CCL5,FOS
Glutathione Redox Reactions I	MGST1
Role of Osteoblasts in Rheumatoid Arthritis Signaling Pathway	BMP4,IL33,MMP8
Activation of kainate receptors upon glutamate binding	GNB3
Nucleotide catabolism	ENTPD4
IL-27 Signaling Pathway	GADD45G,HLA-A
TR/RXR Activation	SYT12,UCP3
IL-6 Signaling	FOS,IL33
Cardiac conduction	SCN4B,SCN5A
fMLP Signaling in Neutrophils	CHP1,GNB3
G-protein beta:gamma signalling	GNB3
Thrombin signalling through proteinase activated receptors (PARs)	GNB3
TNFR2 Signaling	FOS
Dopamine Degradation	Aldh3b3
Synaptic Long Term Potentiation	CHP1,PPP1R3C
Response to elevated platelet cytosolic Ca2+	AHSG,SERPINA3
HGF Signaling	FOS,ITGB6
GABA Receptor Signaling	ABAT,GNB3
Activation of Matrix Metalloproteinases	MMP8
Signal amplification	GNB3
MAPK targets/ Nuclear events mediated by MAP kinases	FOS
Airway Inflammation in Asthma	IL33
LPS/IL-1 Mediated Inhibition of RXR Function	FMO2,IL33,MGST1
Sperm Motility	GNB3,MERTK,PLAAT1
Antimicrobial peptides	LCN2

Late endosomal microautophagy	HSPA8
DHCR24 Signaling Pathway	AHSG,APOD
Ethanol Degradation II	Aldh3b3
GABAergic Receptor Signaling Pathway (Enhanced)	ABAT,GNB3
Gαi Signaling	APLNR,GNB3
MSP-RON Signaling in Cancer Cells Pathway	FOS,MYC
Transcriptional regulation by the AP-2 (TFAP2) family of transcription factors	MYC
MIF-mediated Glucocorticoid Regulation	CD74
Signaling by Rho Family GTPases	FOS,GNB3,ITGB6
Circadian Rhythm Signaling	GNB3,MYC,PTGDS
Hereditary Breast Cancer Signaling	GADD45B,GADD45G
Noradrenaline and Adrenaline Degradation	Aldh3b3
Complement System	C7
Estrogen Receptor Signaling	FOS,GNB3,MMP8,MYC
G alpha (s) signalling events	GNB3,PDE7A
Glutathione-mediated Detoxification	MGST1
Endocannabinoid Cancer Inhibition Pathway	ATF3,MYC
Inhibition of Matrix Metalloproteases	MMP8
Chronic Myeloid Leukemia Signaling	CHP1,FOS,MYC
Neurotransmitter release cycle	SLC17A7
Pyrimidine Ribonucleotides Interconversion	ENTPD4
Dilated Cardiomyopathy Signaling Pathway	MYH7B,SCN5A
T Cell Exhaustion Signaling Pathway	FOS,HLA-A,HLA-DQA1,HLA-DQB1,HLA-DRB5
PTEN Signaling	CNKSR3,ITGB6
RET signaling	IRS2
Corticotropin Releasing Hormone Signaling	FOS,NR4A1
Type II Diabetes Mellitus Signaling	IRS1,IRS2
Role of NFAT in Regulation of the Immune Response	CD4,CHP1,FOS,GNB3,HLA-A,HLA-DQA1,HLA-DQB1,HLA-DRB5
April Mediated Signaling	FOS
Acetone Degradation I (to Methylglyoxal)	CYP1B1
Necroptosis Signaling Pathway	CHP1,MERTK
Role of Pattern Recognition Receptors in Recognition of Bacteria and Viruses	CCL5,IL33
eNOS Signaling	HSPA1A/HSPA1B,HSPA8
Defensins	CD4
Vasopressin regulates renal water homeostasis via Aquaporins	GNB3
B Cell Activating Factor Signaling	FOS

Pyrimidine Ribonucleotides De Novo Biosynthesis	ENTPD4
Sirtuin Signaling Pathway	GADD45B,GADD45G,MYC
Xenobiotic Metabolism Signaling	CYP1B1,FMO2,MGST1
Arachidonic acid metabolism	CYP1B1
Carboxyterminal post-translational modifications of tubulin	TTLL7
GPER1 signaling	GNB3
PI3K Signaling in B Lymphocytes	ATF3,CHP1,FOS,IRS1,IRS2
Cytosolic sensors of pathogen-associated DNA	IFI16
Estrogen Biosynthesis	CYP1B1
Apelin Pancreas Signaling Pathway	APLNR
Inhibition of ARE-Mediated mRNA Degradation Pathway	ZFP36,ZFP36L1
HOTAIR Regulatory Pathway	MMP8,MYC
Transcriptional Regulatory Network in Embryonic Stem Cells	BMP4,MYC
Phagosome Maturation	HLA-A,HLA-DRB5
Role of IL-17F in Allergic Inflammatory Airway Diseases	CXCL10
iNOS Signaling	FOS
nNOS Signaling in Neurons	CHP1
Dendritic Cell Maturation	HLA-A,HLA-DQA1,HLA-DQB1,HLA-DRB5,IL33
G alpha (z) signalling events	GNB3
HMGB1 Signaling	FOS,IL33
Transcriptional activity of SMAD2/SMAD3:SMAD4 heterotrimer	MYC
TP53 Regulates Transcription of Cell Cycle Genes	PLK2
Androgen Signaling	DNAJB1,GNB3
Role of Osteoclasts in Rheumatoid Arthritis Signaling Pathway	CHP1,FOS,MMP8
Gαq Signaling	CHP1,GNB3
CTLA4 Signaling in Cytotoxic T Lymphocytes	FOS,HLA-A,HLA-DQA1,HLA-DQB1,HLA-DRB5
NR1H2 and NR1H3-mediated signaling	APOD
MYC Mediated Apoptosis Signaling	MYC
FAT10 Cancer Signaling Pathway	ACKR3
Ribonucleotide Reductase Signaling Pathway	FOS,MYC
Autoimmune Thyroid Disease Signaling	HLA-A,HLA-DQA1,HLA-DQB1,HLA-DRB5
Netrin Signaling	CHP1,FMO2
Transcriptional regulation by RUNX2	HAND2
Transcriptional regulation of granulopoiesis	MYC

TNFR1 Signaling	FOS
UVC-Induced MAPK Signaling	FOS
UVB-Induced MAPK Signaling	FOS
Signaling by TGF-beta Receptor Complex	ITGB6
Spliceosomal Cycle	HSPA8
Serotonin Receptor Signaling	GNB3,IL33,MYC,PLAAT1
Role of Cytokines in Mediating Communication between Immune Cells	IL33
Phototransduction Pathway	GNB3
T Helper Cell Differentiation	HLA-A,HLA-DQA1,HLA-DQB1,HLA-DRB5
E3 ubiquitin ligases ubiquitinate target proteins	HLA-A
Folate Signaling Pathway	MYC
Oxidative Ethanol Degradation III	Aldh3b3
EGF Signaling	FOS
Regulation of eIF4 and p70S6K Signaling	IRS1,ITGB6
Neurexins and neuroligins	SYT12
TNF signaling	OTUD1
CD27 Signaling in Lymphocytes	FOS
Role of IL-17A in Arthritis	CCL5
Dopamine-DARPP32 Feedback in cAMP Signaling	CHP1,PPP1R3C
FXR/RXR Activation	IL33,MGST1
Iron uptake and transport	LCN2
Triacylglycerol Degradation	ABHD6
Metabolism of water-soluble vitamins and cofactors	AOX1
Allograft Rejection Signaling	HLA-A,HLA-DQA1,HLA-DQB1,HLA-DRB5
B Cell Development	HLA-A,HLA-DQA1,HLA-DQB1,HLA-DRB5
GABA receptor activation	GNB3
Endometrial Cancer Signaling	MYC
Regulation of the Epithelial Mesenchymal Transition by Growth Factors Pathway	EGR1,FOS
Collagen degradation	MMP8
Primary Immunodeficiency Signaling	CD4
Xenobiotic Metabolism CAR Signaling Pathway	FMO2,MGST1
UFMylation Signaling Pathway	MYC
IL-2 Signaling	FOS
Xenobiotic Metabolism PXR Signaling Pathway	MGST1,PPP1R3C
CCR5 Signaling in Macrophages	CCL5,CD4,FOS,GNB3
Peroxisomal protein import	IDH1
Melatonin Degradation I	CYP1B1
Oxytocin in Brain Signaling Pathway	GNB3,PLAAT1

Adrenomedullin signaling pathway	FOS,IL33
ERB2-ERBB3 Signaling	MYC
Human Embryonic Stem Cell Pluripotency	BMP4,MYC
Immunoregulatory interactions between a Lymphoid and a non-Lymphoid cell	CD300LD,HLA-A
Ephrin Receptor Signaling	GNB3,ITGB6
Type I Diabetes Mellitus Signaling	HLA-A,HLA-DQA1,HLA-DQB1,HLA-DRB5
Degradation of the extracellular matrix	MMP8
Role of PI3K/AKT Signaling in the Pathogenesis of Influenza	CCL5
Coronavirus Pathogenesis Pathway	CCL5,FOS
CD40 Signaling	FOS
Mitotic Roles of Polo-Like Kinase	PLK2
Phospholipases	PLAAT1
Superpathway of Melatonin Degradation	CYP1B1
Cell Cycle: G1/S Checkpoint Regulation	MYC
IL-8 Signaling	FOS,GNB3
TP53 Regulates Transcription of DNA Repair Genes	FOS
Cell surface interactions at the vascular wall	CD74,MERTK
Integrin Signaling	ITGB6,TSPAN4
Translocation of SLC2A4 (GLUT4) to the plasma membrane	TBC1D4
Basal Cell Carcinoma Signaling	BMP4
Ephrin B Signaling	GNB3
Senescence-Associated Secretory Phenotype (SASP)	FOS
Serotonin Degradation	Aldh3b3
trans-Golgi Network Vesicle Budding	HSPA8
Calcium Signaling	CHP1,MYH7B
Macropinocytosis Signaling	ITGB6
Hepatic Cholestasis	CHP1,IL33
Antiproliferative Role of Somatostatin Receptor 2	GNB3
Role of NFAT in Cardiac Hypertrophy	CHP1,GNB3
Signaling by NOTCH1	MYC
NF-κB Activation by Viruses	CD4
Neurotrophin/TRK Signaling	FOS
IL-7 Signaling Pathway	MYC
Renal Cell Carcinoma Signaling	FOS
Glycosaminoglycan metabolism	CHP1
G alpha (12/13) signalling events	GNB3

Dopamine Receptor Signaling	PPP1R3C
EIF2 Signaling	ATF3,MYC
PI Metabolism	GDPD3
Estrogen-Dependent Breast Cancer Signaling	FOS
JAK/STAT Signaling	FOS
BEX2 Signaling Pathway	ITGB6
IL-12 Signaling and Production in Macrophages	APOD,FOS
VEGF Family Ligand-Receptor Interactions	FOS
Integrin cell surface interactions	ITGB6
LPS-stimulated MAPK Signaling	FOS
Neutrophil Extracellular Trap Signaling Pathway	CCL5,CHP1,PLAAT1
Oxidative Stress Induced Senescence	FOS
Platelet homeostasis	GNB3
HIPPO signaling	PPP1R3C
Actin Cytoskeleton Signaling	ITGB6,MYH7B
RAB GEFs exchange GTP for GDP on RABs	DENND4A
Opioid Signalling	GNB3
Regulation of Cellular Mechanics by Calpain Protease	ITGB6
Pancreatic Secretion Signaling Pathway	AMY2A,PPP1R3C
Ceramide Signaling	FOS
BMP signaling pathway	BMP4
Degradation of beta-catenin by the destruction complex	MYC
Docosahexaenoic Acid (DHA) Signaling	PLAAT1,SYT12
Actin Nucleation by ARP-WASP Complex	ITGB6
ERBB Signaling	FOS
Communication between Innate and Adaptive Immune Cells	CCL5,CD4,CXCL10,HLA-A,HLA-DRB5,IL33
Class B/2 (Secretin family receptors)	GNB3
Small Cell Lung Cancer Signaling	MYC
Protein folding	GNB3
UVA-Induced MAPK Signaling	FOS
Apelin Cardiomyocyte Signaling Pathway	APLNR
RAF/MAP kinase cascade	IRS1,IRS2
Deubiquitination	IL33,MYC
S Phase	MYC
Neuropathic Pain Signaling in Dorsal Horn Neurons	FOS
Phase I - Functionalization of compounds	CYP1B1
Potassium Channels	GNB3
Response of EIF2AK4 (GCN2) to amino acid deficiency	ATF3

Sumoylation Pathway	FOS
Extracellular matrix organization	ITGB6
Paxillin Signaling	ITGB6
Telomerase Signaling	MYC
α-Adrenergic Signaling	GNB3
Kinetochore Metaphase Signaling Pathway	PPP1R3C
O-linked glycosylation	GCNT1
Antioxidant Action of Vitamin C	PLAAT1
Role of MAPK Signaling in Promoting the Pathogenesis of Influenza	PLAAT1
Regulation of Actin-based Motility by Rho	ITGB6
G Protein Signaling Mediated by Tubby	GNB3,MERTK,MYC
PAK Signaling	ITGB6
IL-15 Production	MERTK
Role of NANOG in Mammalian Embryonic Stem Cell Pluripotency	BMP4
Glioma Signaling	IDH1
Gαs Signaling	GNB3
ROBO SLIT Signaling Pathway	FOS
14-3-3-mediated Signaling	FOS
Endocannabinoid Developing Neuron Pathway	GNB3
Autism Signaling Pathway	HLA-A,IL33
Electron transport, ATP synthesis, and heat production by uncoupling proteins	UCP3
Interleukin-1 family signaling	IL33
G Beta Gamma Signaling	GNB3
Mitotic G1 phase and G1/S transition	MYC
Synaptogenesis Signaling Pathway	HSPA8,SYT12
CCR3 Signaling in Eosinophils	GNB3
Complement cascade	C7
STAT3 Pathway	MYC
RAC Signaling	ITGB6
Iron homeostasis signaling pathway	BMP4
Gap Junction Signaling	HSPA8,MYC
Adipogenesis pathway	BMP4
CGAS-STING Signaling Pathway	IL33
Xenobiotic Metabolism General Signaling Pathway	MGST1
C-type lectin receptors (CLRs)	CLEC10A
Systemic Lupus Erythematosus in B Cell Signaling Pathway	CHP1,FOS,IL33,MYC

PTEN Regulation	EGR1
Cellular Effects of Sildenafil (Viagra)	MYH7B
Semaphorin Neuronal Repulsive Signaling Pathway	ITGB6
Mitochondrial Dysfunction	CHP1,MGST1
Ovarian Cancer Signaling	EDNRA
Microautophagy Signaling Pathway	HSPA8
Glioblastoma Multiforme Signaling	MYC
WNT/β-catenin Signaling	MYC
Protein Sorting Signaling Pathway	SCARB2
D-myo-inositol (1,4,5,6)-Tetrakisphosphate Biosynthesis	PPP1R3C
D-myo-inositol (3,4,5,6)-tetrakisphosphate Biosynthesis	PPP1R3C
Ion channel transport	ANO5
CLEAR Signaling Pathway	CHP1
Leukocyte Extravasation Signaling	MMP8
Synaptic Long Term Depression	PLAAT1
Adrenergic Receptor Signaling Pathway (Enhanced)	IL33
Orexin Signaling Pathway	GNB3
PPARα/RXRα Activation	IRS1
TCF dependent signaling in response to WNT	MYC
Generic Transcription Pathway	NR4A1,ZNF697
Fc epsilon receptor (FCERI) signaling	FOS
Processing of Capped Intron-Containing Pre-mRNA	HSPA8
Neddylation	ANKRD9
Class I MHC mediated antigen processing and presentation	HLA-A,LNX1
Cytotoxic T Lymphocyte-mediated Apoptosis of Target Cells	HLA-A
IL-15 Signaling	MYC
Thrombin Signaling	GNB3
p70S6K Signaling	IRS1
mTOR Signaling	IRS1
Systemic Lupus Erythematosus Signaling	C7,FOS,HLA-A,IL33
Phospholipase C Signaling	CHP1,GNB3,ITGB6
Altered T Cell and B Cell Signaling in Rheumatoid Arthritis	HLA-A,HLA-DQA1,HLA-DQB1,HLA-DRB5,IL33
Regulation of IL-2 Expression in Activated and Anergic T Lymphocytes	CHP1,FOS
Hematopoiesis from Pluripotent Stem Cells	CD4

D-myo-inositol-5-phosphate Metabolism	PPP1R3C
Superpathway of Inositol Phosphate Compounds	PPP1R3C
3-phosphoinositide Degradation	PPP1R3C
3-phosphoinositide Biosynthesis	PPP1R3C
Regulation of the Epithelial-Mesenchymal Transition Pathway	EGR1
TEC Kinase Signaling	FOS,GNB3,ITGB6
SAPK/JNK Signaling	GNB3,IRS1
PI3K/AKT Signaling	ITGB6
B Cell Receptor Signaling	EGR1
NF-κB Signaling	BMP4,IL33
Osteoarthritis Pathway	ITGB6

Table S2. - Cardiac Function in Prolyl-4-Hydroxylase $1^{-/-}$ Mice at 30 Days Post-MI Surgery

Cardiac function 30 days post-MI	WTMI	PHD1$^{-/-}$MI	p Value
Ejection fraction, %	43.11 ± 3.48	54.17 ± 1.52	0.0054
Fractional shortening, %	21.32 ± 1.96	27.85 ± 0.95	0.0049
Left ventricular internal dimension at systole, mm	3.57 ±0.23	3.114 ±0.14	0.089
Left ventricular internal dimension at diastole, mm	4.53 ±0.25	4.31 ±0.18	0.48
Left ventricular volume at systole, mL	55.66 ±8.01	39.48 ±3.77	0.062
Left ventricular volume at diastole, mL	95.77 ± 13.10	85.60 ±7.49	0.48
Stroke volume, mL	30.33 ±2.37	49.90 ± 1.80	0.0001
Cardiac output, mL/min	15.14 ± 1.64	25.04 ± 1.09	0.0010
Heart rate, BPM	479.3 ±25.33	477.2 ± 17.12	0.95
E/A Ratio	2.06± 0.32	2.14± 0.35	0.88

Data presented as mean ± SEM.
